# A comparative cross-reactivity and paraspecific neutralization study on *Hypnale hypnale*, *Echis carinatus*, *and Daboia russelii* monovalent and therapeutic polyvalent anti-venoms

**DOI:** 10.1371/journal.pntd.0010292

**Published:** 2022-03-28

**Authors:** Vaddaragudisalu D. Sandesha, Bhaskar Darshan, Chandrashekar Tejas, Kesturu S. Girish, Kemparaju Kempaiah

**Affiliations:** 1 Department of Studies in Biochemistry, University of Mysore, Manasagangotri, Mysuru, Karnataka, India; 2 Department of Studies and Research in Biochemistry, Tumkur University, Tumakuru, Karnataka, India; Liverpool School of Tropical Medicine, UNITED KINGDOM

## Abstract

Envenoming by the hump-nosed pit viper (*Hypnale hypnale*) raises concern as it inflicts significant debilitation and death in the Western Ghats of India and in the adjacent island nation of Sri Lanka. In India, its medical significance was realized only during 2007 due to its misidentification as *Echis carinatus* and sometimes as *Daboia russelii*. Of late, several case reports have underlined the ineptness of the existing polyvalent anti-venom therapy against *H*. *hypnale* envenoming. Currently, *H*. *hypnale* bite has remained dreadful in India due to the lack of neutralizing anti-venom therapy. Hence, this study was undertaken to establish a systematic comparative, biochemical, pathological, and immunological properties of Sri Lankan *H*. *hypnale* venom alongside Indian *E*. *carinatus*, and *D*. *russelii* venoms. All three venoms differed markedly in the extent of biochemical activities including proteolytic, deoxyribonuclease, L-amino acid oxidase, 5’-nucleotidase, hyaluronidase, and indirect hemolytic activities. The venoms also differed markedly in their pathological properties such as edema, hemorrhage, myotoxic, cardiotoxic, and coagulant activities. The venoms showed stark differences in their protein banding pattern. Strikingly, the affinity-purified rabbit monovalent anti-venoms prepared against *H*. *hypnale*, *E*. *carinatus*, and *D*. *russelii* venoms readily reacted and neutralized the biochemical and pathological properties of their respective venoms, but they insignificantly cross-reacted with, and thus failed to show paraspecific neutralization of any of the effects of the other two venoms, demonstrating the large degree of variations between these venoms. Further, the Indian therapeutic polyvalent anti-venoms from VINS Bioproducts, and Bharath Serums and Vaccines failed to protect *H*. *hypnale* venom-induced lethal effects in mice.

## Author summary

In India, the ‘Big four’ snakes, viz. *Naja naja*, *Bungarus caeruleus*, *Daboia russelii*, and *Echis carinatus* are considered medically significant. Hence therapeutic polyvalent anti-venoms are prepared against the concoction of the venoms from said snakes. In India, the dreadful effects of *Hypnale hypnale* bite was realized only during 2007 due to its misidentification as *Echis carinatus* and or *Daboia russelii* bite. Several case reports from India and Sri Lanka have underscored the ineptness of Indian therapeutic anti-venoms against *H*. *hypnale* envenoming. As *H*. *hypnale* venom is not marketed in India, Sri Lankan *H*. *hypnale* venom was compared with Indian *E*. *carinatus*, and *D*. *russelii* venoms for their biochemical, pathological, and immunological properties. The venoms differed greatly in the said properties. The rabbit monovalent anti-venoms of *H*. *hypnale*, *E*. *carinatus*, and *D*. *russelii* venoms neutralized the activities of their venoms, but they insignificantly cross-reacted with, and failed to show paraspecific neutralization of the effects of the other two venoms. Further, the therapeutic polyvalent anti-venoms from VINS, and Bharat failed to neutralize the *H*. *hypnale* venom-induced lethal toxicity in mice. Thus, this study is pressing the need for suitable anti-venom to treat the dreadful *H*. *hypnale* bite in India.

## Introduction

Snakebite is still a largely ignored public health crisis despite the World Health Organization (WHO) declaring it as a neglected tropical disease. Globally, snakebite kills around 81000 to 138000 people each year, and about thrice the victims suffer from permanent physical disability and disfigurement [1]. The impoverished rural populations of Asia, Africa, and Latin America are severely affected [2,3]. Basically, India is an agrarian nation as over half of its population earns its livelihood through farming and agriculture. Therefore, snake-human conflicts are expected to be high. With an annual death rate of about 58,000 and the disability rate of about 140,000, India is the global hotspot of snakebite [1]. As of now, the so-called big-four snakes, common spectacled cobra (*Naja naja)*, common krait *(Bungarus caeruleus)*, Russell`s viper *(Daboia russelii)*, and saw-scaled viper *(Echis carinatus)* are the medically focused species in the country. This is based on their relatively dense distribution across the wide geographic area. Hence, the therapeutic anti-venoms are made against the concoction of venoms of the said four species. Though polyvalent anti-venom therapy is available, the high casualty rate in India is likely due to either the difficulty in accessing, or the poor success rate of anti-venom therapy, or the envenoming by other medically important but, overlooked venomous snakes. Incidentally in a recent study, Senji Laxme et al. [4] described a high degree of variability in the composition, biochemical and pathological effects, and toxicity profiles of venoms from the neglected relatives of the big-four species. They are *Naja kaouthia* (Arunachal Pradesh, and West Bengal), *Bungarus fasciatus* (West Bengal), *Bungarus sindanus*, and *Echis carinatus sochureki* (Rajasthan). The study highlighted a markedly reduced cross-neutralizing competence of four commercial Indian anti-venoms against the said neglected snake venoms [4]. Similarly, with its rich biodiversity and region-specific ecosystem, the venomous snake hump-nosed pit viper (*Hypnale hypnale*) is densely distributed in the Western Ghats region (Kerala) of India, and also in the neighboring island nation of Sri Lanka [5,6]. *H*. *hypnale* bite is known to cause life-threatening systemic complications, such as hemorrhage, coagulopathy, fibrinolysis, thrombocytopenia, severe bleeding, and acute renal failure. Besides, it causes debilitating tissue necrosis at the bite site [7,8]. Until recent past, its dreadful venomous bite was misidentified as *E*. *carinatus*, or sometimes as *D*. *russelii* bite as these three vipers share close physical resemblance [5]. Considering its fairly wide distribution and severity of the bite, WHO in 2010 scheduled the *H*. *hypnale* as a category I snake of medical relevance [9]. It was Sri Lankan medical personnel who strongly emphasized the pharmacotherapeutic ineffectiveness of the imported Indian polyvalent anti-venom (from Haffkine’s Institute), especially against *H*. *hypnale* envenomation. However, of late, the Indian medical personnel too have realized the inaptness [5,10,11]. Currently, sincere efforts are in progress to integrate *H*. *hypnale* venom into the anti-venom manufacturing regimen in Sri Lanka [12]. Unfortunately, the Indian counterpart is yet to realize the importance of specific anti-venom to treat *H*. *hypnale* bite. Therefore, in the absence of an effective anti-venom therapy, the *H*. *hypnale* envenoming has remained disastrous and plagued with human sufferings. Hence, there is a pressing need for the appropriate therapeutic anti-venom. Thus, in the current study, a more systematic, and comparative investigation has been undertaken to demonstrate the extent of cross-reactivity, and paraspecific neutralization of Sri Lankan *H*. *hypnale* venom with the Indian *E*. *carinatus*, and *D*. *russelii* venoms against their respective monovalent anti-venoms and commercial therapeutic polyvalent anti-venoms.

## Materials and methods

### Ethics statement

All the experiments were approved by the Institutional Human Ethical Committee (IHEC-UOM No. 70/Res/2020–21), University of Mysore, Mysuru and conducted in accordance with the ethical guidelines.

All animal experiments were approved by the Institutional Animal Ethical Committee (UOM/IAEC/04/2020), Department of Studies in Zoology, University of Mysore, Mysuru, and were in accordance with the guidelines of the Committee for the Purpose of Control and Supervision of Experiments on Animals (CPCSEA).

### Chemicals and reagents

Protein-A agarose, goat anti-rabbit IgG, 3, 3′, 5, 5′-tetramethylbenzidine (TMB), immobilon-P PVDF membrane, luminol, p-coumaric acid, Freund’s complete and incomplete adjuvants, testicular hyaluronidase, hyaluronic acid, o-dianisidine, horseradish peroxidase (HRP) 250U, and all chemicals were obtained from Sigma, St Louis, USA. Sodium chloride, bovine serum albumin, Folin-Ciocalteu reagent, Tris-HCl, casein, gelatin, trichloroacetic acid, sodium carbonate, calcium chloride, magnesium chloride, acrylamide and bis-acrylamide, sodium dodecyl sulfate (SDS), ammonium persulfate, tetramethyl ethylenediamine (TEMED), sodium formate, triton X-100, Alcian blue, acetic acid, triethanolamine, L-leucine, methanol, and all other chemicals were purchased from Sisco Research Laboratories (SRL), Mumbai, India. HRP-conjugated goat anti-horse IgG (H+L) was purchased from KINESISDx, South Paseo Dr, Brea, CA 90603, USA. Molecular weight markers were obtained from Genetix Biotech. Asia Pvt. Ltd. Bengaluru, India. LDH, CK, and CK-MB kits were purchased from AGAPPE Diagnostics Ltd. Kerala, India. Liquicelin-E and Uniplastin reagents were purchased from TULIP Diagnostics (P) Ltd. India.

### Snake venoms and anti-venoms

The Principal Chief Conservator of Forests (Wildlife) & Chief Wildlife Warden, Karnataka State Forest Department, Govt of Karnataka, (Wildlife permission No. PCCF (WL)/E2/CR-08/2019-20) for the permission to procure and utilize the venoms for the research purpose. *Hypnale hypnale* venom (*Hh*v) was purchased from Latoxan laboratory, (Lot. No. 317.081 & Product ID; L1602) France, as it is not marketed in India. The venom is a pool, obtained from several specimens collected in Sri Lanka. *Daboia russelii* venom (*Dr*v), and *Echis carinatus* venom (*Ec*v) were purchased from Haffkine’s Institute, Mumbai, India, and the venoms are a pool obtained from several specimens collected in different regions of Maharashtra, India. The following poly-specific anti-venoms were used: (a) Snake venom anti-venom (India) from VINS Bioproducts Ltd. (batch number 01AS14075, expiry date: 04/21 was used for all neutralization studies, as the anti-venom got exhausted, the new batch of anti-venom, batch number 01AS21057, expiry date: 07/25 was procured to repeat Western blot experiment during the revision of the manuscript as suggested by the reviewer); (b) Snake venom anti-venom (India) from Bharat Serums and Vaccines (Batch number: A05320019, expiry date: 05/24).

### Collection of human blood

Human blood was collected from the antecubital veins of healthy adult volunteers who were provided with written informed consent, as per the guidelines of the Institutional Human Ethical Committee (IHEC), University of Mysore (UOM), Mysuru.

### Protein estimation and dilution of venoms and anti-venoms

The protein content was estimated according to the method of Lowry *et al*. using Bovine Serum Albumin (BSA) as standard [13]. The affinity-purified monovalent anti-venoms *H*. *hypnale* anti-venom (*Hh*AV), *E*. *carinatus* anti-venom (*Ec*AV), and *D*. *russelii* anti-venom (*Dr*AV) were further diluted independently to 10 mg/ml stock in phosphate-buffered saline (10 mM PBS pH 7.4). The therapeutic polyvalent anti-venoms were dissolved in sterile water as per the manufacturer’s instruction and the estimated yield was 10 mg/ml for BhAV and 7.5 mg/ml for ViAV. The lyophilized *Hh*v, *Ec*v, and *Dr*v samples were made into 10 mg/ml stock in PBS.

### SDS-polyacrylamide gel electrophoresis

The venom samples were subjected to SDS-PAGE (10% and 12.5%, under both non-reducing and reducing conditions) according to the method of Laemmli to obtain protein banding patterns of *Hh*v, *Ec*v, and *Dr*v (25 μg each) using Bio-Rad (Mini-PROTEAN Tetra Cell) unit [14]. After electrophoresis, the gels were stained with 0.25% Coomassie Brilliant Blue R-250, and proteins were visualized after destaining. Further, the images were scanned by using HP Scanjet (Model-G2410).

### Substrate-gel assays

Casein, gelatin, and hyaluronic acid zymography assays were performed independently by incorporating 0.2% each of casein, and gelatin, and 0.017% of hyaluronic acid into 10% polyacrylamide gels. The venoms, *Hh*v, *Ec*v, and *Dr*v, 5–30 μg each for caseinolytic and gelatinolytic activities, and 50 μg each for hyaluronidase activity were loaded onto SDS-PAGE under non-reducing conditions. After electrophoresis, casein/gelatin zymogram was washed with 10 mM sodium phosphate buffer (pH 7.6) containing 2.5% Triton X-100 for about one hour with three washings at 20 min intervals, followed by water wash to remove Triton X-100. Then gels were incubated overnight with 50 mM Tris-HCl buffer pH 7.6 containing 10 mM CaCl_2_ and 150 mM NaCl at 37°C. Finally, the gels were stained with Coomassie Brilliant Blue R-250. For hyaluronidase activity, the gel was soaked consecutively 3 times in 50 ml of 0.1 M sodium phosphate buffer pH 5.8 containing 0.15 M NaCl, 2.5% Triton X-100 for 1 h. This was followed by equilibrating the gel in 0.1 M sodium formate buffer pH 5.0 containing 0.15 M NaCl for 20 h at 37°C with constant agitation. The gel was washed in 0.015 M Tris-HCl buffer pH 7.9 and placed in Alcian blue staining solution. In all three cases, the clear zones against the blue background of undigested respective substrates indicated the enzyme activities of venoms [15,16]. Further, the images were scanned by using HP Scanjet (Model-G2410).

### Proteolytic activity

The proteolytic activity was determined as described by Satake *et al*. [17]. Briefly, 50 μg/ml each of *Hh*v, *Ec*v, and *Dr*v were independently incubated with 0.4 ml of casein (2%) in 0.2 M Tris–HCl buffer pH 8.5 with the final reaction volume of 1 ml at 37°C for 2.5 h. The reaction was stopped by adding 1.5 ml 0.44 M trichloroacetic acid, kept for 30 min, and centrifuged at 90 × g for 15 min. Further 1 ml supernatant was mixed with 2.5 ml 0.4 M sodium carbonate solution and 0.5 ml Folin-Ciocalteau reagent (1:2, v/v), allowed to stand for 30 min at room temperature, and the absorbance was measured at 660 nm. The activity was expressed in units, where one unit was defined as the amount of venom required to increase the absorbance by 0.01 OD at 660 nm/min at 37°C. For *in vitro* neutralization studies, *Hh*v (50 μg/ml), *Ec*v (50 μg/ml), and *Dr*v (100 μg/ml) were independently pre-incubated with various doses (250–5000 μg/ml) of anti-venoms (*Hh*AV/*Ec*AV/*Dr*AV/BhAV/ViAV) for 15 min at room temperature. Then, the proteolytic activity was assayed by adding 1 ml of buffered substrate solution as described above. Respective venoms alone were served as the control experiments.

### Deoxyribonuclease activity

Deoxyribonuclease (DNase) activity was determined by performing agarose gel electrophoresis. Briefly, 50 μg of *Hh*v, *Ec*v, and *Dr*v were independently incubated with 250 ng of calf thymus DNA for 60 min, at 37°C in a final volume of 30 μl (PBS). The reaction mixture was subjected to electrophoresis on 1.2% agarose gel at 50 V in TAE buffer (40 mM Tris-base and 1 mM EDTA, pH 8.0) for 1 h. DNase 1 (10 U) was used as a positive control. After electrophoresis, the gel was visualized and photographed on an ultraviolet transilluminator (Alliance 2.7, Uvitech) [18].

### Hemolytic activity

Hemolytic activity was determined according to the method of Boman & Kaletta [19] with minor modifications using washed human erythrocytes. Briefly, for indirect hemolytic activity, packed human erythrocytes and egg yolk suspended in 10 mM PBS pH 7.4 (ratio1:1:8; v/v) was used. For direct hemolytic activity, 1 ml of the packed erythrocytes were taken and made up to 10 mL in PBS. In either case, 1 ml of the suspension was incubated independently with 10 μg/ml each of *Hh*v, *Ec*v, and *Dr*v for 1 h at 37 ^0^C. The reaction was stopped by adding 9 mL of ice-cold PBS and centrifuged at 1000 x g for 10 min at 4 ^0^C. The amount of hemoglobin released in the supernatant was measured at 540 nm. The activity was expressed as a percent of hemolysis against 100% lysis of cells by water. For *in vitro* neutralization studies, 20 μg/ml each of *Hh*v, *Ec*v, and *Dr*v were independently pre-incubated with various doses (100–2000 μg/ml) of anti-venoms (*Hh*AV/*Ec*AV/*Dr*AV/BhAV/ViAV) for 15 min at room temperature. Then, the respective hemolytic activities were assayed independently by adding 1 ml of respective erythrocytes suspensions as described above. Respective venoms alone were served as control experiments.

### L-Amino acid oxidase activity

L-Amino acid oxidase activity was determined by the method of Tan and Tan [20] with little modifications. The reaction volume 1 ml contained 50 μl peroxidase (0.007%, 510 NIH units/mg), 0.1% L-leucine and 0.0065% o-dianisidine in 0.2 M triethanolamine buffer pH 7.6 and incubated for 3 min at room temperature. Then, 50 μg/ml each of *Hh*v, *Ec*v, and *Dr*v were added independently, and an increase in the absorbance was monitored at 440 nm. One unit of activity was defined as the amount of enzyme required to cause an increase in O.D. by 0.001 at 440 nm/min. For *in vitro* neutralization studies, 100 μg/ml each of *Hh*v, *Ec*v, and *Dr*v were independently pre-incubated with various doses (500–10000 μg/ml) of anti-venoms (*Hh*AV/*Ec*AV/*Dr*AV/BhAV/ViAV) for 15 min at room temperature. Then, the L-Amino acid oxidase activity was assayed by adding 1 ml of the reaction mixture as described above. Respective venoms alone were served as the control experiments.

### 5’-Nucleotidase activity

The 5’-Nucleotidase activity was determined by the method of Avruch and Wallach [21] with minor modifications. The reaction volume of 1 ml contained 10 mM MgCl_2_, 50 mM NaCl, 10 mM KCl, 50 mM Tris-HCl buffer pH 7.4, and 10 mM AMP were incubated independently with *Hh*v (15 μg/ml), *Ec*v (30 μg/ml), and *Dr*v (40 μg/ml) for 30 min at 37 ^0^C. The ascorbic acid method [22] was used to determine the released inorganic phosphate. A measure of 1 ml of ascorbic acid reagent, containing equal parts of 0.42% ammonium molybdate in 1 N sulfuric acid, 10% ascorbic acid, and water was added to the reaction mixture. Then, the reaction mixture was kept at room temperature for 30 min and the absorbance was monitored at 660 nm. This was quantified by comparison with the reference curve established with KH_2_PO_4_. One unit of 5’-nucleotidase activity was expressed in terms of the release of inorganic phosphorus in μmoles/min/μg. For *in vitro* neutralization studies, *Hh*v (15 μg/ml), *Ec*v (30 μg/ml), and *Dr*v (40 μg/ml) were independently pre-incubated with various doses (75–1500 μg/ml) of anti-venoms (*Hh*AV/*Ec*AV/*Dr*AV/BhAV/ViAV) for 15 min at room temperature. Then, the 5’-Nucleotidase activity was assayed by adding 1 ml of the reaction mixture as described above. Respective venoms alone were served as the control experiments.

### Plasma re-calcification time

The plasma coagulant activity was determined according to the method of Condrea *et al*. [23]. Briefly, fresh healthy human blood was mixed with 3.2% trisodium citrate in the ratio 9:1 (v/v). The blood was centrifuged for 15 min at 500 x g. The obtained supernatant was used as platelet-poor plasma (PPP), which was pre-warmed to 37 ^0^C before use. PPP, 0.2 ml was mixed with 10 μl of Tris-HCl buffer (10 mM, pH 7.4) and incubated at 37 ^0^C for 1 min. *Hh*v, *Ec*v, and *Dr*v, 10 μg/ml each were added independently into PPP, followed by quick addition of 20 μL of 0.25 M CaCl_2_. The clotting time was recorded in seconds against a light source. The normal clotting time of PPP was noted by adding 20 μl of CaCl_2_. For *in vitro* neutralization studies, *Hh*v, *Ec*v, and *Dr*v, 20 μg/ml each were pre-incubated with various doses (100–2000 μg/ml) of anti-venoms (*Hh*AV/*Ec*AV/*Dr*AV/BhAV/ViAV) for 15 min at room temperature. Then, the coagulant activity was determined by adding PPP as described above. Respective venoms alone were served as control experiments.

### Activated partial thromboplastin time and prothrombin time

*Hh*v, *Ec*v, and *Dr*v (5 μg/ml) were independently pre-incubated with PPP (100 μl) for 1 min at 37 ^0^C. For activated partial thromboplastin time (APTT) assay, 100 μl reagent (LIQUICELIN-E, phospholipids preparation derived from rabbit brain with ellagic acid) was added and incubated for 3 min at 37 ^0^C. The clotting was initiated by adding 100 μl of 0.02 M CaCl_2_ and the clotting time was recorded in seconds. For the prothrombin time (PT) assay, the clotting was initiated by adding 200 μl of the PT reagent (UNIPLASTIN, rabbit brain thromboplastin). The time taken for the formation of a visible clot was recorded in seconds. The APTT ratio and the international normalized ratio (INR) for PT at each point were calculated from the values of control plasma incubated with the buffer for an identical period. For *in vitro* neutralization studies, 20 μg/ml each of *Hh*v, *Ec*v, and *Dr*v were independently pre-incubated with various doses (100–2000 μg/ml) of anti-venoms (*Hh*AV/*Ec*AV/*Dr*AV/BhAV/ViAV) for 15 min at room temperature. Then, APTT and PT assays were performed as described above by adding PPP. Respective venoms alone were served as control experiments [24].

### Thrombin-like activity

The thrombin-like activity was determined according to the method described by Denson with minor modifications [25]. Briefly, 100 μl of reaction mixture containing human fibrinogen (3 mg/mL), 10 mM NaCl in 10 mM Tris–HCl buffer pH 7.6 was used. The clot formation was initiated independently by adding 10 μg/ml of *Hh*v, *Ec*v, and *Dr*v. The clotting time was recorded in seconds. The fibrinogen alone in Tris-HCl buffer served as the negative control and 10 U of thrombin served as a positive control. For *in vitro* neutralization studies, 20 μg/ml each of *Hh*v, *Ec*v, and *Dr*v were independently pre-incubated with various doses (100–2000 μg/ml) of anti-venoms (*Hh*AV/*Ec*AV/*Dr*AV/BhAV/ViAV) for 15 min at room temperature. Then, the thrombin-like activity was measured by adding the reaction mixture as described above. Respective venoms alone were served as control experiments.

### Fibrinogenolytic activity

The fibrinogenolytic activity was performed according to the method described by Ouyang *et al*. [26]. Briefly, various amounts of *Hh*v, *Ec*v, and *Dr*v were independently incubated with fibrinogen (50 μg) for 60 min at 37 ^0^C in a 40 μl reaction mixture containing 10 mM NaCl and 10 μl of (10 mM) Tris-HCl buffer pH 7.6. Then, 20 μl of denaturing buffer (0.5 M Tris-HCl, pH 6.8, 1 M urea, 4% SDS, and 4% β-mercaptoethanol) was added and analyzed on 10% SDS-PAGE. The banding pattern was visualized by staining with Coomassie Brilliant Blue R-250 [27]. Further, the images were scanned by using HP Scanjet (Model-G2410).

### Fibrinolytic activity

The normal human citrated blood was centrifuged for 15 min at 500 x g to separate PPP. Then, 100 μl PPP was mixed with an equal volume of 0.025 M CaCl_2_ for 30 min at 37 ^0^C to get the soft fibrin clot. The fibrin clot was washed thoroughly 5–6 times with 10 mM PBS pH 7.6. The washed fibrin clot was independently incubated with 5 and 10 μg each of *Hh*v, *Ec*v, and *Dr*v in a final volume of 40 μl reaction mixture containing 10 mM Tris–HCl buffer pH 7.6 at 37 ^0^C for 5 h. The reaction was stopped by adding 20 μL of sample buffer containing 4% SDS, 1 M urea, and 4% β-mercaptoethanol. The samples were boiled for 3 min and centrifuged to settle down the debris of the plasma clot. An aliquot of 3 μl supernatant was analyzed in 7.5% SDS-PAGE for fibrin degradation products [27]. Further, the images were scanned by using HP Scanjet (Model-G2410).

### Experimental animals

Swiss albino mice (20–25 g) were collected from the University Central Animal Facility and housed in specific pathogen-free conditions, with water and food. The New Zealand white female rabbits, 6 months old, weighing 1.5–2.0 kg were obtained from the Department of Livestock Production and Management, Veterinary College, Bengaluru, India, and housed in specific pathogen-free conditions, with water and food in University Central Animal Facility.

### Murine model of edema-inducing activity

The edema-inducing activity was assayed according to the method of Yamakawa *et al*. [28]. Briefly, groups (n = 3) of mice were independently injected with various doses of *Hh*v, *Ec*v, and *Dr*v in 20 μl PBS into right footpads, the left footpads were injected with 20 μl PBS which served as negative controls. After an hour, mice were euthanized using over dose (5–10 mg/kg i.p.) of xylazine, legs were dissected off at the ankle joint and weighed. An increase in weight due to edema was calculated as edema ratio, which equals the weight of the edematous leg × 100/weight of the negative control leg. The minimum edematic dose (MED) was defined as the amount of venom required to cause the edema ratio of 120%. For *in vivo* neutralization studies, *Hh*v (5 μg), *Ec*v (5 μg), and *Dr*v (10 μg) were independently pre-incubated with various doses (50–500 μg) of anti-venoms (*Hh*AV/*Ec*AV/*Dr*AV/BhAV/ViAV) for 15 min at room temperature prior to administration as above. Further, the photographs of mice legs were captured by using iPhone 11 Pro (Model-A2215).

### Murine model of hemorrhagic activity

The hemorrhagic activity was assayed as described by Kondo *et al*. [29]. Different doses of *Hh*v, *Ec*v, and *Dr*v in 30 μl of PBS were independently injected intradermally into the mice skin (n = 3). The group of mice that received PBS alone served as a negative control. After 3 h, the mice were euthanized using over dose (5–10 mg/kg i.p.) of xylazine, and the dorsal patch of the skin was carefully removed and observed for hemorrhage in the inner surface of the skin against PBS injected control mice skin. The diameter of the hemorrhagic spot on the inner surface of the skin was measured in mm^2^. MHD (minimum hemorrhagic dose) was defined as the minimum dose of venom that is required to produce a 10 mm^2^ diameter of hemorrhagic spot. For *in vivo* neutralization studies, 5 μg each of *Hh*v, *Ec*v, and *Dr*v were independently pre-incubated with various doses (50–500 μg) of anti-venoms (*Hh*AV/*Ec*AV/*Dr*AV/BhAV/ViAV) for 15 min at room temperature prior to administration as above. Further, the photographs of mice skin were captured by using iPhone 11 Pro (Model-A2215).

### Determination of lethality of venoms using a murine model

The mean lethal dose of venom at which 50% of the test animals die (LD_50_) was determined using a group of 10 mice weighing 20–25 g. The venom was injected through the intraperitoneal (i.p.) route with the corresponding doses ranging from 1 to 10 mg/kg body weight in 0.1 ml of PBS. The symptoms and signs of toxicity were observed and the survival time of each animal was recorded for 24 h. Finally, the LD_50_ value was determined according to the mathematical scheme of Meier and Theakston [30]. Independent experiments were performed for *Hh*v, *Ec*v, and *Dr*v.

### Murine model of venom-induced tail tissue destruction

The groups of mice (n = 3) were injected subcutaneously with LD_50_ doses of *Hh*v (3.5 mg/kg), *Ec*v (2.5 mg/kg), and *Dr*v (3.0 mg/kg) in 50 μl PBS pH 7.4 into the mice tail that is 3 cm from the tip of the tail. Mice injected with 50 μl of PBS served as a control group. The severity of the tail injury was judged visually and scored according to a 10-point scale; 0 = no injury, 1 = edema, 2 = edema with minor hemorrhage, 4 = edema with hemorrhage causing less than 25% tail discolouration, 6 = edema, and major hemorrhage or wound causing 25–50% tail discolouration, 8 = edema, and major hemorrhage or wound causing 50–75% tail discolouration, 10 = edema, and major hemorrhage or wound causing more than 75% tail discolouration. The tail injury observations were recorded for 2 days after venom injection [18]. Further, the photographs of mice tail were captured by using iPhone 11 Pro (Model-A2215).

### Murine model of myotoxicity and cardiotoxicity

Myotoxicity was determined according to the method of Gutierrez *et al*. [31]. The cytoplasmic marker enzymes, lactate dehydrogenase (LDH), and creatine kinase (CK) levels were determined in the serum of mice. The groups of mice (n = 4) were independently injected intramuscularly (i.m.) into the thigh muscles with the LD_50_ doses of each venom, *Hh*v (3.5 mg/kg), *Ec*v (2.5 mg/kg), and *Dr*v (3.0 mg/kg) in 50 μl PBS. The groups of mice receiving 50 μl PBS alone were served as control experiments. After 24 h, mice were anaesthetized using xylazine (1–4 mg/kg i.p.) and blood was drawn by cardiac puncture. The obtained serum was assayed for LDH, CK, and CK-MB activities using AGAPPE diagnostic kits. Activities were expressed as units/L. For *in vivo* neutralization studies, post 10 min of venom injection, ED_50_ of *Hh*AV (140 mg/kg) and *Ec*AV, *Dr*AV, BhAV, and ViAV, 700 mg/kg were injected intravenously (i.v.) into the mice tail. After 24 h mice were anesthetized, and blood was drawn to prepare serum. Then, the assay was performed as described above.

### Immunization of rabbits, preparation of anti-venoms, and affinity purification of IgG fraction

Immunization of rabbits and purification of antibodies was performed as described by Shashidharamurthy *et al*. [32]. Briefly, 100 μg each of *Hh*v, *Ec*v, and *Dr*v were independently dissolved in 100 μl PBS (10 mM, pH 7.4), mixed thoroughly with an equal volume of Freund’s complete adjuvant, and injected intradermally (i.d.) at different sites (40–50 μl at 4–5 sites) in the back of female rabbits. Three booster doses of venoms were administered at the same dose but with an equal volume of Freund’s incomplete adjuvant at weekly intervals. About 15 ml of blood was drawn from the marginal ear vein on the 9^th^ day after the third booster dose and allowed to coagulate for 24 h at 8–10 ^0^C to obtain the anti-serum. In each case, about 10 ml of the anti-serum was subjected to ammonium sulfate precipitation to obtain the crude immunoglobulin G fraction, which was subjected to protein-A agarose affinity column chromatography. The column was equilibrated with PBS and loaded with 5 mg of crude immunoglobulin G fraction in 2 ml of PBS. The elution was carried out using 0.2 M glycine-HCl buffer, pH 2.9. Aliquots, 1 ml were collected and pooled after reading the optical density at 280 nm and then neutralized using 1 M Tris–HCl buffer pH 8.0. Samples were further subjected to dialysis using a 3.4 kDa membrane against PBS for 24 h at 4 ^0^C. Thus, the obtained monovalent anti-venoms were designated as *H*. *hypnale* anti-venom (*Hh*AV), *E*. *carinatus* anti-venom (*Ec*AV), and *D*. *russelli* anti-venom (*Dr*AV) and were used for the neutralization study.

### Western blotting

Briefly, 25 μg each of *Hh*v, *Ec*v, *Dr*v, and BSA were subjected to SDS-PAGE (10%, non-reducing) independently according to the method of Laemmli [14]. After electrophoresis, the proteins were transferred to the PVDF membrane using a transfer unit (Bio-Rad Mini-PROTEAN Tetra Cell) containing 0.12 M Tris-glycine transfer buffer pH 8.3. Blotting was carried out for 90 min at 100 V at 4 ^0^C. To check the extent of protein transferred to the PVDF membrane, Ponceau-S reversible stain was used. Membranes were blocked with TBST (10 mM Tris-HCl buffer pH 8.0 containing 150 mM NaCl and 0.05% Tween-20) containing 5% non-fat milk powder + 1% BSA for 1 h. After blocking, the blots were washed (3–4 times) with a wash buffer (TBST) and followed by the incubation with primary antibodies *Hh*AV/*Ec*AV/*Dr*AV/BhAV/ViAV/pre-immune rabbit serum, (dilution; 1: 20,000) for 3 h at room temperature. After washing (3–4 times) with TBST, the blots were incubated with horseradish peroxidase (HRP) conjugated secondary antibody (1: 10,000 dilutions; goat anti-rabbit/goat anti-equine) for 1 h at room temperature. Then the blots were washed (3–4 times) with washing buffer (TBST) and developed using an enhanced chemiluminescence method and visualized using a chemiluminescence system (Bio-Rad ChemiDoc-MP, USA).

### Enzyme-linked immunosorbent assay (ELISA)

Briefly, 96 well titer plates were independently coated with 100 ng of *Hh*v, *Ec*v, and *Dr*v (venom/buffer, w/v) prepared with 0.2 M carbonate-bicarbonate buffer pH 9.6 and incubated overnight at 4 ^0^C. For blank, wells were coated with 100 μl of blocking buffer (0.2 M carbonate-bicarbonate buffer, pH 9.6 containing 5% skimmed milk + 1% BSA). Plates were washed after each stage, using 5–6 changes of wash buffer-PBST (10 mM PBS, pH 7.4 containing 0.2% Tween-20). Then plates were incubated with a blocking buffer at room temperature for 1 h to block non-specific reactivity. The anti-serum/anti-venom (*Hh*AV/*Ec*AV/*Dr*AV/BhAV/ViAV) was taken at an initial concentration of 1 mg/ml in PBS, and subsequent dilutions were added at an initial dilution of 1:5, followed by increments of 1:5 serial dilutions in PBS. The wells were washed with PBST followed by the addition of 100 μl of diluted anti-venoms to each well and incubated overnight at 4 ^0^C. Then, plates were washed and incubated with secondary antibody (1:10,000 dilutions in PBS, v/v) conjugated with horseradish peroxidase for 1 h at room temperature. Then, plates were washed and 100 μl of undiluted chromogenic substrate TMB was added to each well and incubated at room temperature for 30 min. The reaction was stopped by adding 50 μl 1N H_2_SO_4_. The color developed was read at 405 nm using a Biotek ELx 800-ELISA plate reader, and subsequently, titer values for anti-serums/anti-venoms (*Hh*AV/*Ec*AV/*Dr*AV/BhAV/ViAV) were calculated [33,34].

### Determination of median effective dose (ED_50_) of anti-venoms using a murine model

Seven groups of mice (n = 6) were independently injected with various doses (70–700 mg/kg) of *Hh*AV/*Ec*AV/*Dr*AV/BhAV/ViAV through tail vein (i.v.) post 10 min of *H*. *hypnale* venom (2 LD_50_; 7 mg/kg) injection (i.p.). Mice were kept under observation for 24 h and the time of death was recorded. The experiment was performed independently by injecting 2 LD_50_ of *Ec*v (5 mg/kg), and *Dr*v (6 mg/kg) also. Independent groups of mice that were injected with 2 LD_50_ of *Hh*v, *Ec*v, and *Dr*v alone, and a group of mice that received PBS alone were served as control experiments. The percent survival analysis of mice was done by constructing the Kaplan-Meier survival curve, the *p*-value was calculated using the log-rank (Mantel-Cox) test [18]. The neutralizing ability of anti-venom was expressed as the Median Effective Dose (ED_50_), i.e., the venom/anti-venom ratio at which half of the population of injected mice is protected. Venoms were injected through the intraperitoneal (i.p.) route. If injected through the intravenous (i.v.) route, animals would die before injecting lifesaving anti-venoms. Intraperitoneal (i.p.) route of venom injection has been practiced in our laboratory/Department, and as well as in other laboratories [35,36].

### Statistical analysis

The results were expressed as mean ± SEM of three independent experiments. Statistical significance was determined using one-way/ two-way ANOVA, followed by Bonferroni post-test, as required. Significance was accepted at *p*>0.05 (ns), *p*< 0.05 (*), *p* < 0.01 (**), *p*< 0.001 (***), and *p* < 0.0001 (****). Data were analyzed using the statistical package GraphPad Prism (GraphPad Software 8.0, USA). Confidence intervals (95%) to the effective dose (ED) of anti-venoms were calculated using Microsoft Excel (Ver. 2019, USA).

## Results and discussion

### Comparative biochemical properties of *H*. *hypnale*, *E*. *carinatus*, and *D*. *russelii* venoms

**Fig 1:**
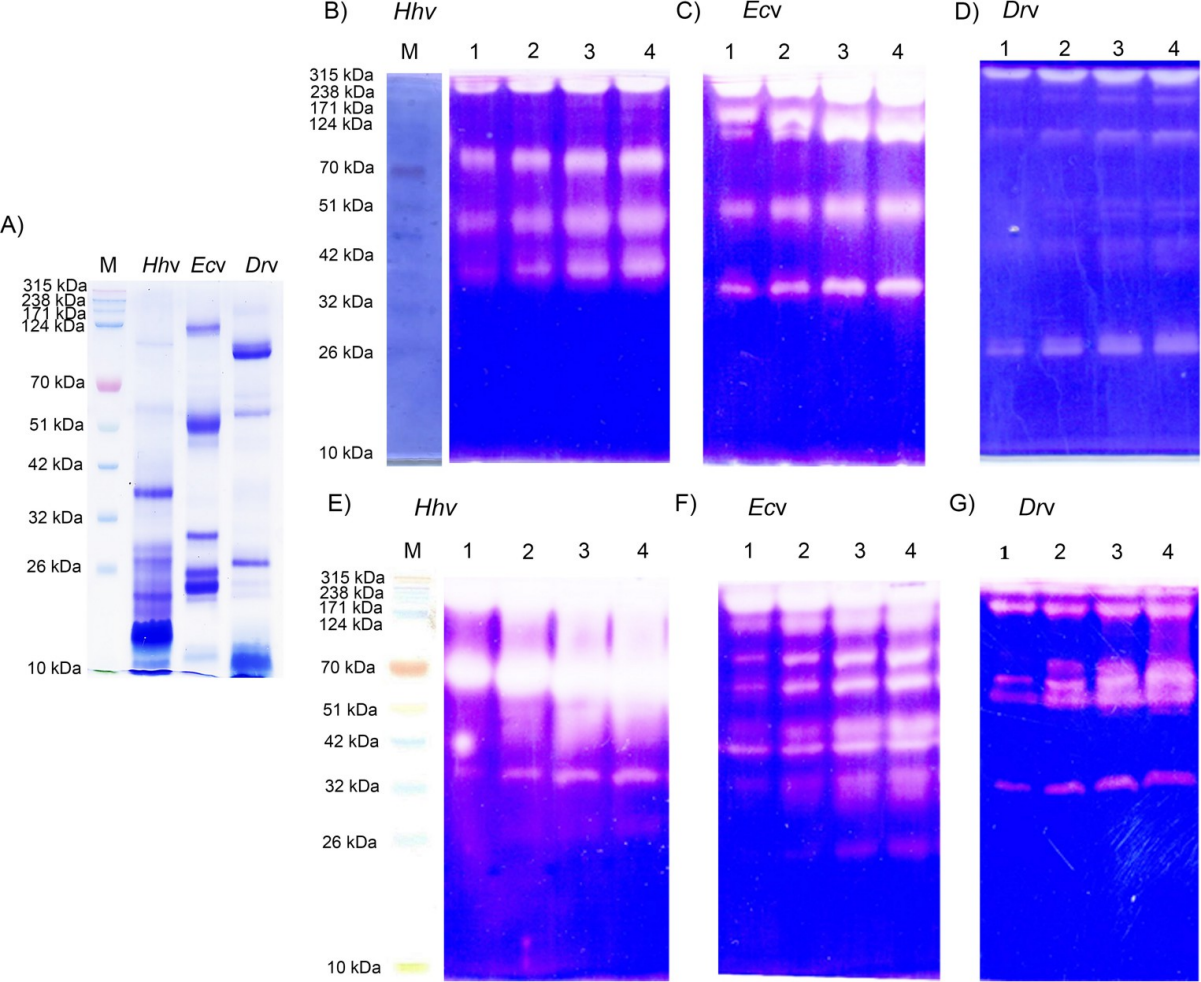
SDS-PAGE pattern, and zymography of *H*. *hypnale*, *E*. *carinatus*, and D. *russelii* venoms. (A) SDS-PAGE (10%) banding pattern of venoms; *Hh*v, *Ec*v, and *Dr*v (25 μg each) were used and analyzed under non-reducing condition. For zymography, the venoms *Hh*v, *Ec*v, and *Dr*v were independently studied for proteolytic activity banding patterns in substrate gel assays. B-D and E-G respectively represent caseinolytic and gelatinolytic activities of three venoms. The substrates, casein, and gelatin (0.2%) were incorporated into respective gels (10%). The SDS-PAGE was performed under non-reduced condition. In lanes 1–4 in respective gels, different doses, 5, 10, 20, and 30 μg of venoms were used. M represents the molecular weight markers in kDa. The clear translucent zones against a blue background indicate the respective activities in gels. The images were captured by using HP Scanjet (Model-G2410).

Envenoming by the pit viper, *H*. *hypnale* is as severe as the envenoming by the other vipers like *E*. *carinatus*, and *D*. *russelii* in the Indian subcontinent [5,37,38]. *H*. *hypnale* is endemic to the Western Ghats of India, and the island nation of Sri Lanka, while *E*. *carinatus* and *D*. *russelii* are endemic to the subcontinent [39]. The venoms of these three vipers predominantly disturb the hemostatic system leading to fatal bleeding complications. In addition, they are also known to inflict devastating tissue necrosis at the bite site. Hence, in this study, *H*. *hypnale* venom was comparatively studied for its biochemical, pathological, and immunological properties with the venoms of *E*. *carinatus*, and *D*. *russelii*. As the venoms of the latter two species are intensely studied in many laboratories [12,40–43], in this study, their properties were compared only wherever necessary. All three venoms displayed a unique protein-banding pattern in SDS-PAGE (10%) under non-reduced condition. Medium and low molecular weight protein bands were prominently seen in *H*. *hypnale* venom as compared to *E*. *carinatus* and *D*. *russelli* venoms which displayed a wide range, large, medium, and small molecular weight protein bands (Fig 1A). The corresponding densitometric scanning analysis of protein bands of each venom is provided in figure (S1 Fig). Further, when the venoms were also resolved in 10% and 12.5% gel (SDS-PAGE under both reduced and non-reduced conditions), none of the three venoms revealed conspicuous protein bands of molecular mass range less than 10 kDa (S1C and S1D Fig). Disintegrins (5–8 kDa) are abundant in viperid venoms [44], however, in our study, SDS-PAGE did not reveal their existence. Detailed LC-MS/MS study would reveal their existence in these three venoms. However, the recent proteomics study using the venom sample collected from a single Indian *H*. *hypnale* by Vanuopadath et al. [45] revealed 37 proteins belonging to nine different enzymatic and non-enzymatic protein families. They include serine proteases, metalloproteases, phospholipases A_2_, thrombin-like enzymes, phospholipase B, C-type lectins/snacles, disintegrins, cysteine-rich secretary proteins, and nerve growth factor. Further, extensive proteomics study on Sri Lankan *H*. *hypnale* venom revealed to contain more or less similar protein families, but with kallikrein and L-amino acid oxidase enzymes additionally [46–48]. In our study, the *H*. *hypnale*, *E*. *carinatus*, and *D*. *russelii* venoms differed markedly in caseinolytic (Fig 1B–1D) and gelatinolytic (Fig 1E–1G) activity banding patterns in zymography. The caseinolytic and gelatinolytic zymograms of these venoms along with negative control BSA and positive control trypsin are given in the figure (S2 Fig). *H*. *hypnale* venom revealed a high content of proteolytic activity as evidenced by the intense translucent caseinolytic and gelatinolytic activity bands over the other two venoms in respective gels. *H*. *hypnale* venom hydrolyzed the gelatin readily over casein as evidenced by the relatively intense activity bands in gelatin zymogram compared to casein zymogram. However, the estimated caseinolytic activity of *H*. *hypnale* venom was found to be higher, about 3 and 50 times respectively than *E*. *carinatus*, and *D*. *russelli* venoms (Table 1). This endorses the intense proteolytic activity bands observed for *H*. *hypnale* venom compared to *E*. *carinatus* and *D*. *russelii* venoms in casein and gelatin zymograms. Thus, the estimated caseinolytic activity of these venoms varied as *H*. *hypnale* > *E*. *carinatus* > *D*. *russelii* venoms. Several studies reported the extensive proteolytic activity (both serine, and metallo) from these three venoms [9,49,50]. *H*. *hypnale* venom differed in its hyaluronidase activity banding pattern in zymography as it revealed distinct activity bands in around 70 kDa and 20 kDa regions, while *E*. *carinatus* and *D*. *russelli* venoms revealed the activity bands in regions around 70 kDa and above (S3 Fig). Thus, these two venoms are likely to share similar hyaluronidase activity. As a ‘spreading factor’, hyaluronidase activity was found to participate strongly in both local and systemic toxicities of snake venoms [51]. Further, *H*. *hypnale* venom showed strong 5’-nucleotidase activity, and the activity varied as *H*. *hypnale* >*D*. *russelli* >*E*. *carinatus* venoms. The enzyme was found to be associated with anti-coagulant activity [22]. All three venoms appear to share nearly similar indirect hemolytic activity which corresponds to phospholipase A_2_ (PLA_2_) activity of snake venoms. PLA_2_ enzymes represent one of the major families of enzymatic toxins of snake venoms. Interestingly, most PLA_2_ enzymes are multifunctional in nature, they were known for inducing multiple pathological properties (Imitate whole venom pathology) in addition to catalytic activity. Hence, they were the molecules of special focus and subjected for extensive structure-function relationship studies [52–54]. They were isolated and extensively characterized from snake venoms, including *H*. *hypnale*, *E*. *carinatus*, and *D*. *russelii* venoms [55–57]. Similarly, *H*. *hypnale*, *E*. *carinatus*, and *D*. *russelii* venoms revealed nearly similar L-amino acid oxidase activity. L-amino acid oxidases were known to exert various biological and pathological effects, such as platelet aggregation, hemorrhage, and cytotoxicity, and also induction of apoptosis [58]. The quantitative proteolytic, indirect hemolytic, 5’-nucleotidase, and L-amino acid oxidase activities were summarized in Table 1. Strikingly, all three venoms did not degrade calf thymus DNA (S4 Fig), thus exhibiting a high degree of similarity by lacking deoxyribonuclease (DNase) activity. However, in a recent study, Senji Laxme et al. [59] have demonstrated an exceptionally high DNase activity in venom samples of *D*. *russelii* from the Punjab region while the venom samples from the other geographical regions of India recorded low to negligible DNase activity. DNase activity was found to be critically implicated in both systemic and local toxicities of snake venoms. Inhibition of DNase activity of *N*. *naja* venom significantly increased the survival time in mice, while the addition of DNase-I to *E*. *carinatus* venom greatly augmented the lethal potency, and reduced the extent of local toxicity of the venom [18]. Thus, it is interesting and important to explore the possible DNase activity of *H*. *hypnale* and *E*. *carinatus* venoms from different geographic origins [60].

**Table 1:** Protease activity; one unit of activity is defined as the amount of enzyme required to cause an increase in O.D. by 0.01 at 660 nm/min (a). Indirect hemolytic activity; it is expressed as a percent of hemolysis (b). Direct hemolytic activity; it is expressed as a percent of hemolysis (c). L-amino acid oxidase activity; one unit of activity is defined as the amount of enzyme required to cause an increase in O.D. by 0.001 at 440 nm/min (d). 5’-nucleotidase activity; one unit of activity is expressed in terms of the release of inorganic phosphorus in μ moles/min/μg of protein (e). The data is presented as Mean ± SEM (n = 3).

Souce of venom	Protease activity ^(a)^ (Units/mg)	Indirect hemolytic activity ^(b)^ (%)	Direct hemolytic activity ^(c)^ (%)	L-amino acid oxidase assay ^(d)^ (Units/μg)	5’-nucleotidase activity ^(e)^ (μmoles/min/μg of protein)
*H*. *hypnale*	5300 ± 35	95.26 ± 1.5	-	7.83 ± 0.12	326.42 ± 0.92
*D*. *russelii*	100 ± 6	97.77 ± 0.23	-	7.33 ± 0.3	168.41 ± 0.516
*E*. *carinatus*	1800 ± 12	92.17 ± 1.17	-	8.67 ± 0.33	74.62 ± 0.41

### Comparative pathological properties of *H*. *hypnale*, *E*. *carinatus*, and *D*. *russelii* venoms

**Fig 2:**
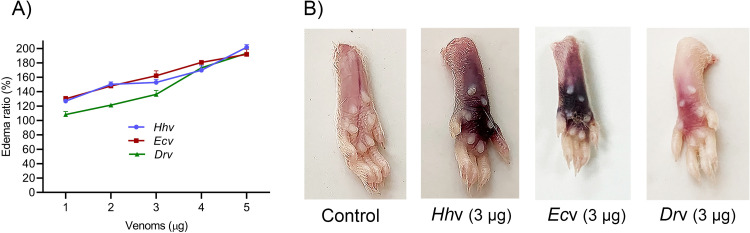
Murine model of edema-inducing activity of *H*. *hypnale*, *E*. *carinatus*, and *D*. *russelii* venoms. (A) Dose-dependent edema-inducing activity of *Hh*v, *Ec*v, and *Dr*v. Different doses of venoms (1–5 μg) in 20 μl PBS were injected into the intraplantar surface of mice right footpads. The left footpads that received PBS alone were served as negative controls. After 1 h of injection, mice were euthanized using xylazine; both the legs were removed at the ankle joint and weighed. An increase in weight due to edema was calculated as the edema ratio, which equals the weight of the edematous leg × 100/weight of the negative control leg. (B) Mice foot pads showing hemorrhagic (*Hh*v and *Ec*v) and non-hemorrhagic (*Dr*v) edema. The data is presented as Mean ± SEM (n = 3).

**Fig 3:**
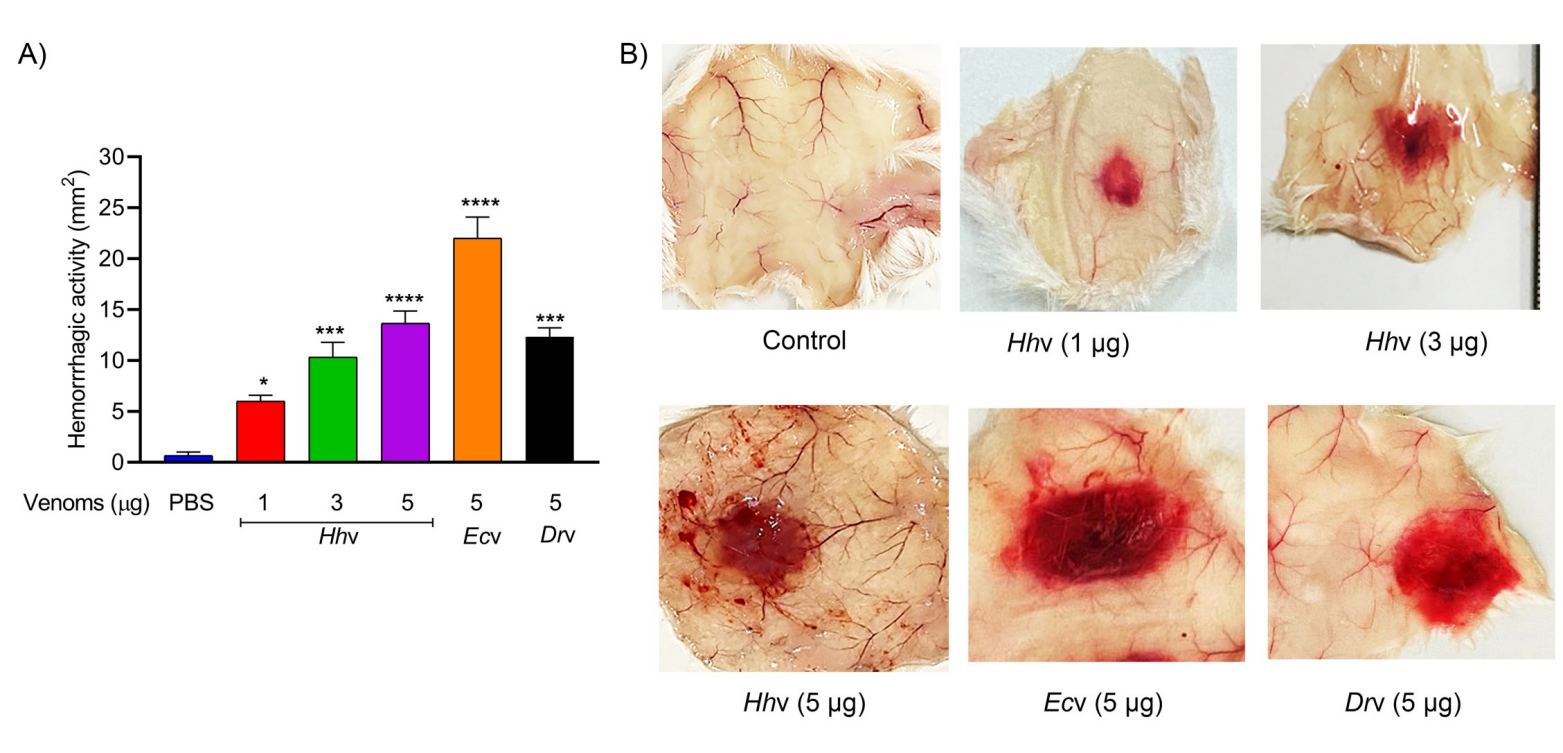
Murine model of hemorrhagic activity of *H*. *hypnale*, *E*. *carinatus*, and *D*. *russelii* venoms. (A) Groups of mice were independently injected intradermally with different doses, 1–5 μg of *Hh*v, and 5 μg each of *Ec*v and *Dr*v in 30 μl PBS respectively. The group of mice were injected with PBS served as a negative control. After 3 h of injection, mice were euthanized using xylazine and removed skin at the venom injected spot, the area of hemorrhagic spots that appeared on the inner surface of the skin was measured in mm^2^. (B) The inner surface of the skin tissues shows hemorrhagic spots. The data is presented as Mean ± SEM (n = 3) and analyzed using one-way/two-way ANOVA followed by Bonferroni post-tests, ‘**** *p* <0.0001, *** *p* <0.001, ** *p* <0.01, * *p* <0.05, and ns (not significant) >0.05. ‘*’ significant compared to the control group (PBS).

**Fig 4:**
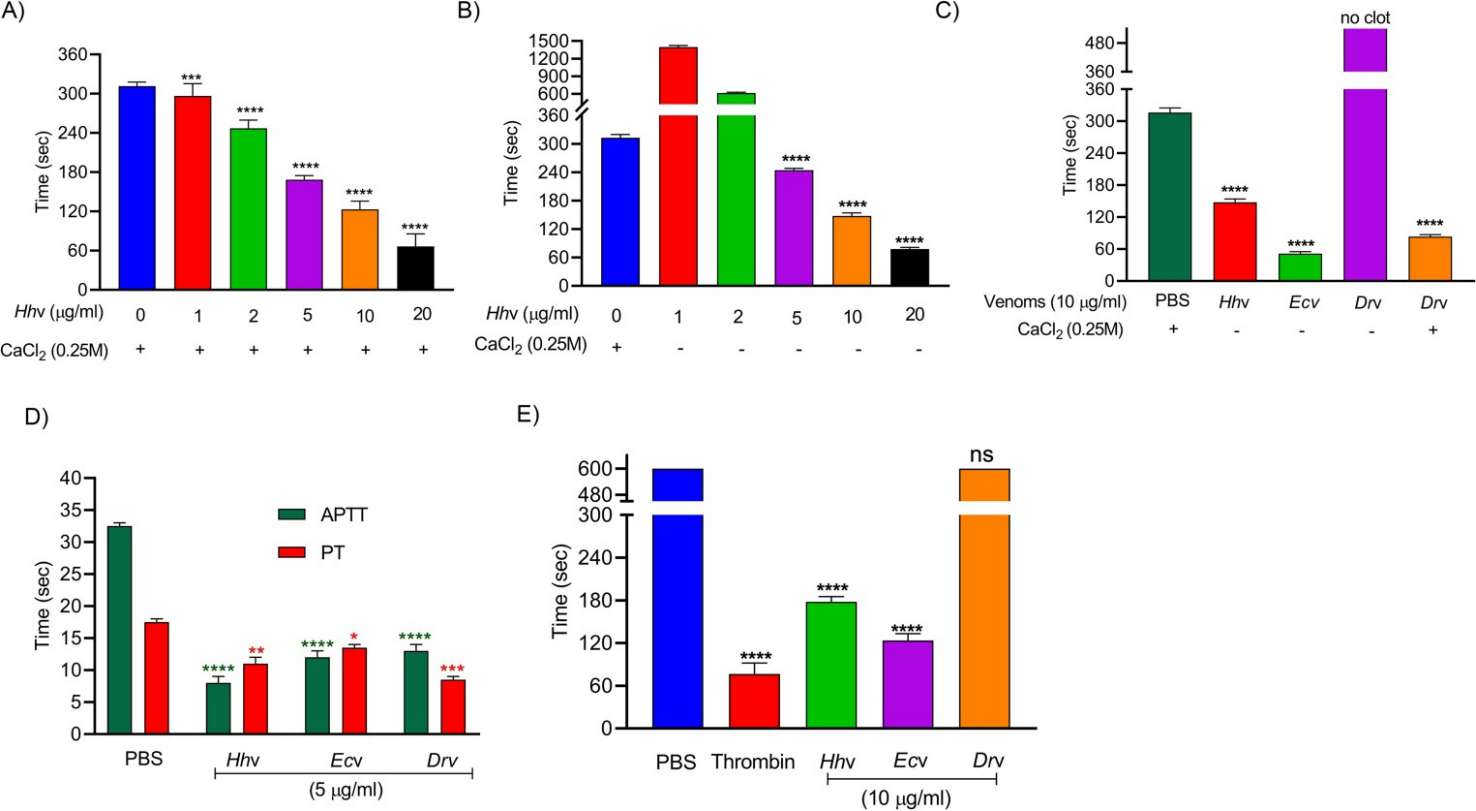
Effect of *H*. *hypnale*, *E*. *carinatus*, and *D*. *russelii* venoms on coagulant activities. (A) Plasma re-calcification time of *Hh*v; 200 μl of citrated human plasma was treated with 1–20 μg/ml of venom for 1 min at 37 ^0^C, clotting was initiated by adding 20 μl 0.25 M CaCl_2._ (B) *Hh*v induced the plasma coagulation in the absence of CaCl_2_; clotting was initiated by adding 1–20 μg/ml of *Hh*v to the 200 μl of citrated human plasma. (C) Comparative plasma re-calcification time of *Hh*v, *Ec*v, and *Dr*v; 200 μl of citrated human plasma was independently treated with 10 μg/ml each of venoms to initiate the clotting. For *Dr*v, clotting was initiated separately by adding 20 μl 0.25 M CaCl_2_. (D) Activated partial thromboplastin time (APTT); 100 μl of citrated human plasma was treated independently with 5 μg/ml each of *Hh*v, *Ec*v, and *Dr*v for 1 min at 37 ^0^C, then 100 μl of reagent (LIQUICELIN-E phospholipids preparation derived from rabbit brain with ellagic acid) was added and incubated for 3 min at 37 ^0^C. The clotting was initiated by adding 100 μl 0.02 M CaCl_2_. Prothrombin time (PT); 100 μl of citrated human plasma was treated independently with 5 μg/ml each of *Hh*v, *Ec*v, and *Dr*v for 1 min at 37 ^0^C. The clotting was initiated by adding 100 μl of PT reagent (UNIPLASTIN–rabbit brain thromboplastin). In all the cases, the plasma devoid of venom was served as control experiments. (E) Thrombin clotting time (TCT); 100 μl of fibrinogen (3 mg/ml) in 10 mM Tris-HCl buffer pH 7.6 was independently treated with 10 μg/ml each of *Hh*v, *Ec*v, and *Dr*v to initiate the clotting. Thrombin, 10 U was used as a positive control, and fibrinogen alone was served as a negative control. In all the above experiments, the time taken for the visible clot formation was recorded in seconds. In all the cases, the data is presented as Mean ± SEM (n = 4) and analyzed using one-way/two-way ANOVA followed by Bonferroni post-tests, ‘**** *p* <0.0001, *** *p* <0.001, ** *p* <0.01, * *p* <0.05, and ns (not significant) >0.05. ‘*’ significant compared to the control group (PBS).

**Fig 5:**
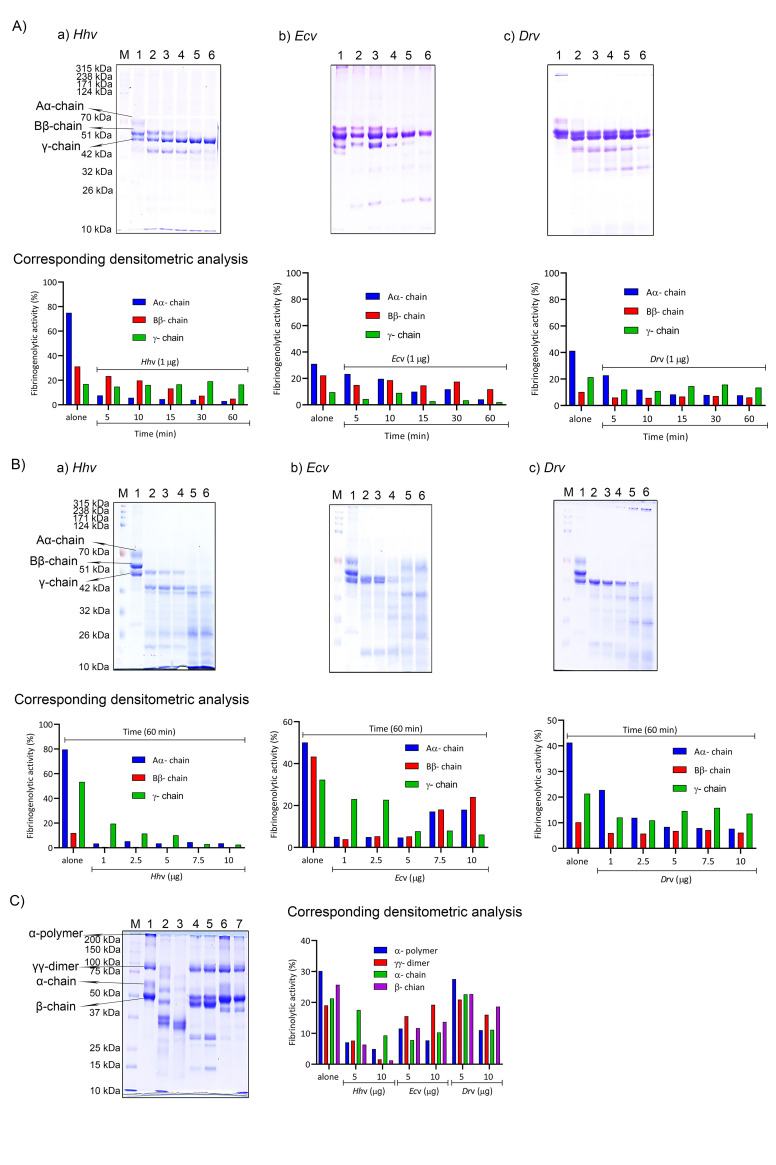
Fibrinogenolytic and fibrinolytic activities of *H*. *hypnale*, *E*. *carinatus*, and *D*. *russelii* venoms. (A) Fibrinogenolytic activity of venoms (time-dependent); fibrinogen, 50 μg was independently incubated with 1 μg each of *Hh*v, *Ec*v, and *Dr*v for different time intervals at 37 ^0^C. Lane I fibrinogen (50 μg) alone, lanes 2–6 where 50 μg of fibrinogen was incubated with *Hh*v (Aa), *Ec*v (Ab), and *Dr*v (Ac) for 5, 10, 15, 30, and 60 minutes respectively. (B) Fibrinogenolytic activity of venoms (dose-dependent); fibrinogen, 50 μg was independently incubated with different doses of *Hh*v, *Ec*v, and *Dr*v for 60 min at 37 ^0^C. Lane I fibrinogen (50 μg) alone, lanes 2–6 where 50 μg of fibrinogen was treated with 1, 2.5, 5, 7.5, and 10 μg of *Hh*v (Ba), *Ec*v (Bb), and *Dr*v (Bc) respectively. (C) Fibrinolytic activity of venoms; 100 μl of washed plasma clot was independently incubated for 16 hours at 37 ^0^C with 5 & 10 μg each of *Hh*v (lanes 2 and 3), *Ec*v (lanes 4 and 5), and *Dr*v (lanes 6 and 7) respectively, and fibrin clot alone (lane 1). In all cases, M represents the molecular weight protein markers in kDa and are analyzed on SDS PAGE (10% for fibrinogenolytic activity and 7.5% for fibrinolytic activity) under reduced condition. The images were captured by using HP Scanjet (Model-G2410). The quantitative degradation patterns of fibrinogen, and fibrin by the *Hh*v, *Ec*v, and *Dr*v are represented through corresponding densitogram images (ImageJ Software Ver. 1.53k, USA) in respective cases.

**Fig 6:**
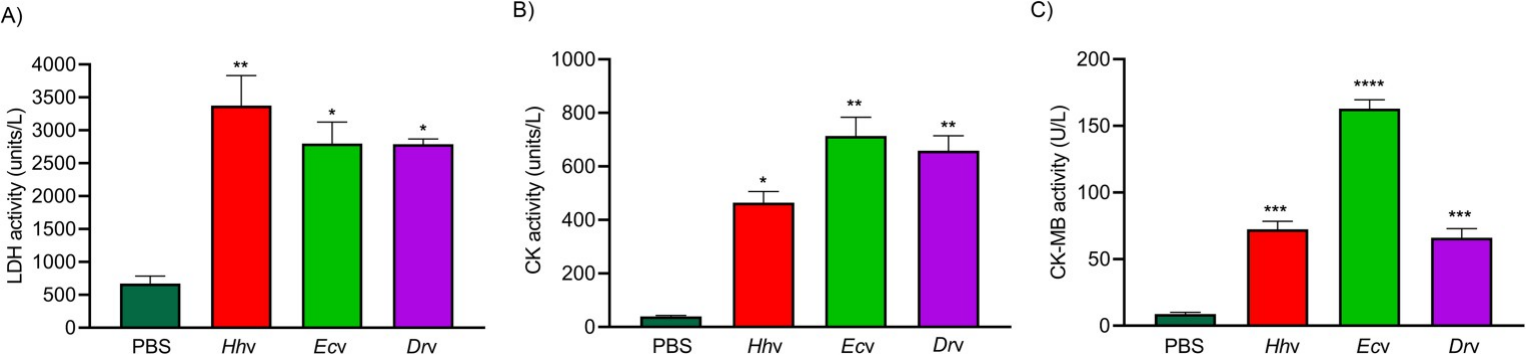
Murine model of myotoxicity and cardiotoxicity of *H*. *hypnale*, *E*. *carinatus*, and *D*. *russelii* venoms. The groups (n = 4) of mice were independently injected intramuscularly (i.m.) with the LD_50_ doses of *Hh*v (3.5 mg/kg), *Ec*v (2.5 mg/kg), and *Dr*v (3.0 mg/kg) into the thigh muscle in 50 μl of PBS. The groups of mice that received PBS alone served as control experiments. After 24 hours, mice were anesthetized using xylazine; blood was drawn by cardiac puncture. The marker enzymes, LDH (A), CK (B), and CK-MB (C) activities (Units/L) were assayed in the mice serum. The data is presented as Mean ± SEM (n = 4) and analyzed using one-way/two-way ANOVA followed by Bonferroni post-tests, ‘**** *p* <0.0001, *** *p* <0.001, ** *p* <0.01, * *p* <0.05, and ns (not significant)>0.05. ‘*’ significant compared to the control group (PBS).

*H*. *hypnale* bite is found to cause severe toxic effects including systemic and local effects. The local effects include edema, hemorrhage, severe pain, blisters, and finally results in necrosis at the bite site. The systemic effects include fatal hemostatic dysfunction leading to disseminated intravascular coagulopathy which results in spontaneous bleeding, pulmonary hemorrhage, and acute kidney injury [5,39,61–63]. In this study, *H*. *hypnale* venom readily induced dose-dependent hemorrhagic edema in the footpads (Fig 2), and hemorrhage in the skin (Fig 3) of Swiss albino mice. The minimum edema dose (MED) and the minimum hemorrhagic dose (MHD) of *H*. *hypnale* venom were found to be 1 μg (95% confidence limits: 0.84–4.12 μg) and 2 μg (95% CL: 0.8–3.58 μg) respectively. *E*. *carinatus* and *D*. *russelii* venoms induced the edema, and hemorrhage with the MED of 0.8 μg (95% CL: 0.6–3.3 μg) and 1 μg (95% CL: 0.49–3.42 μg) and MHD of 1 μg (95% CL: 1.53–5.46 μg) and 3 μg (95% CL: 1.83–6.91 μg) respectively. However, *E*. *carinatus* venom resembles *H*. *hypnale* venom by inducing hemorrhagic edema. The edema and hemorrhage-inducing properties varied as *E*. *carinatus* > *H*. *hypnale* > *D*. *russelli* venoms. Snake venom PLA_2_s are the leading causative agents of edema due to the generation of vasoactive eicosanoids. However, the hemorrhagic edema could be due to the myonecrotic activity or due to hemorrhage intensifying activity of PLA_2_ enzymes [64], or the role of hemorrhagic snake venom metalloproteases (SVMPs) [65]. *H*. *hypnale* venom readily caused necrosis of tissues at the site of injection in a mouse-tail model; similarly, *E*. *carinatus* and *D*. *russelii* venoms also caused the necrosis of tissues. Interestingly, in contrast to *D*. *russelii* venom which did not show any sign of bleeding at the site of injection, both *H*. *hypnale* and *E*. *carinatus* venoms caused prolonged bleeding at the injection site. The bleeding initiated in around 2 hours and continued for about 8 hours post venom injection. All three venoms caused the necrosis of respective tail tissues nearly to a similar extent as the affected tail region gradually turned brittle and showed the signs of dislocation within two days of venom injection (S5 Fig). Although our earlier study attempted to dissect the mechanism of tissue necrosis considering the massive NETosis at the *E*. *carinatus* venom injection site, the mechanism appears much more complex and the role of myonecrotic PLA_2_s, matrix-degrading SVMPs, and hyaluronidases cannot be ignored [18,65,66]. *H*. *hypnale* venom was found to interfere in the clotting time of citrated human plasma [9,63]. In our study too, the venom strongly interfered in the clotting process, it dose-dependently reduced the plasma re-calcification time (Fig 4A–4C), activated partial thromboplastin time (APTT), prothrombin time (PT) (Fig 4D), and thrombin clotting time (TCT) (Fig 4E), suggesting its robust procoagulant nature. Interestingly, it interfered in the clotting of citrated human plasma even in the absence of added CaCl_2_, where it showed biphasic effects. Under low doses, it showed anticoagulant activity, increased the clotting time, while under higher doses, it was pro-coagulant, and reduced the clotting time (Fig 4B). *E*. *carinatus* venom also induced the clotting of citrated human plasma in the absence of CaCl_2_. Thus, the calcium ion independent procoagulant activity of *H*. *hypnale* venom could be similar to that of *E*. *carinatus* venom. Ecarin, a metalloproteinase from *E*. *carinatus* venom, which is a prothrombin activator and catalyzes the formation of thrombin without requiring any cofactors such as Ca^2+^, phospholipid, and factor V [67,68]. Thus, the procoagulant activity of *H*. *hypnale* venom could have been due to the Ecarin-like prothrombin activator. However, *E*. *carinatus* venom also contains a calcium ion-dependent prothrombin activator, Carinactivase [69]. However, the observed biphasic effect on coagulant activity of *H*. *hypnale* venom appears interesting and exciting to explore. Interestingly, in this study, *D*. *russelii* venom differed markedly in TCT, as it did not cause the clotting of the fibrinogen (Fig 4E), suggesting the lack of prothrombin activating and or thrombin-like activities in the venom. However, a thrombin-like serine protease, Russelobin, was isolated from Pakistan`s *D*. *russelii russelii* venom [70]. In addition, factor X activator, RVV-X [71], and prothrombin activating metalloprotease, Rusviprotease [72] were isolated and studied from *D*. *russelii* venom. *H*. *hypnale* venom readily hydrolyzed the fibrinogen, under lower doses, Aα-chain was preferentially hydrolyzed over Bβ-chain, and the ϒ-chain appeared resistant (Fig 5Aa). However, at higher doses, the ϒ-chain was also hydrolyzed by the venom (Fig 5Ba). A similar trend was also observed for the *E*. *carinatus* (Fig 5Ab and 5Bb), and *D*. *russelii* venoms (Fig 5Ac and 5Bc). The corresponding densitograms provide the quantitative measure of the degradation of different chains of fibrinogen in each case. Thus, the effects of *H*. *hypnale* venom on the plasma coagulation process were likely due to strong thrombin-like activity. However, considering the anticoagulant property of *D*. *russelii* venom in TCT (Haffkine venom), the venom might have hydrolyzed the fibrinogen from its c-terminal end, cleaving the D domain which is required for binding to the central E domain during the polymerization process [73]. The *H*. *hypnale* venom efficiently hydrolyzed the fibrin clot as well, the α-polymer, ϒϒ-dimers, α-chain, and β-chain were hydrolyzed. However, the α-polymer was hydrolyzed preferentially over the other chains. In contrast, both *E*. *carinatus*, and *D*. *russelii* venoms readily hydrolyzed the α-chain and α-polymer, but not β-chain and ϒϒ-dimers. The corresponding densitograms provide a measure of quantitative degradation of respective chains (Fig 5C). Thus, *H*. *hypnale* venom efficiently hydrolyzed the fibrin clot over *E*. *carinatus* and *D*. *russelii* venoms. Isolation and detailed characterization of fibrin(ogen)olytic enzyme/s from *H*. *hypnale* venom appears interesting and might lead to developing clinically significant molecules [74]. Thus, this study systematically explored the fibrin(ogen)olytic activity of *H*. *hypnale* venom. However, this activity is very well studied for various other snake venoms [75–77]. *H*. *hypnale* venom readily induced the myotoxicity, it damaged the muscle tissues as evidenced by the elevated levels of cytoplasmic marker enzymes, lactate dehydrogenase (LDH) (Fig 6A), creatine kinase (CK) (Fig 6B), and creatine kinase-MB (CK-MB) (Fig 6C) enzymes in the serum of experimental mice. All three venoms induced the myotoxicity nearly to a similar extent. The myotoxicity of *E*. *carinatus* and *D*. *russelii* venoms was well addressed in several cases [78,79]. However, *E*. *carinatus* venom was found to be more cardiotoxic among the three venoms as it recorded the highest CK-MB activity (Fig 6C). Nevertheless, cardiac troponin and natriuretic peptides are the better markers. *H*. *hypnale* venom was found to be rich in PLA_2_ enzymes, however, the cytolytic/myonecrosis of snake venom PLA_2_s was subjected for intense study [45,46,57,62,80,81]. Though information is inadequate, few studies were attempted to evaluate the myotoxicity of *H*. *hypnale* venom [9,38,82]. In addition, the venom was found to cause acute kidney injury and affected the functioning of heart, lungs, liver, and gastrointestinal tract [9,38,61,62].

### Reactivity/cross-reactivity of *H*. *hypnale*, *E*. *carinatus*, and *D*. *russelii* venoms with the monovalent and therapeutic polyvalent anti-venoms

**Fig 7:**
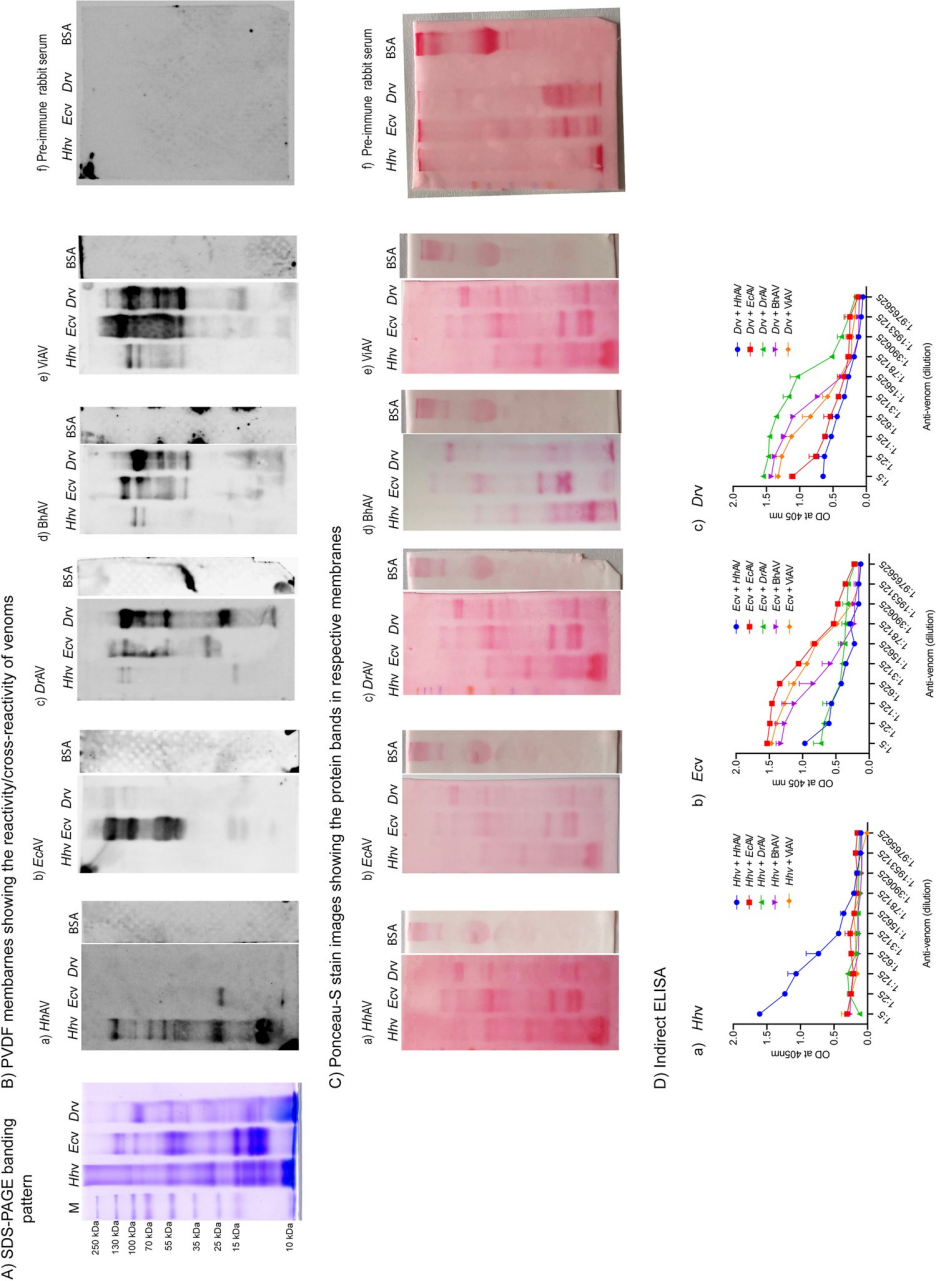
Cross-reactivity of *H*. *hypnale*, *E*. *carinatus*, and *D*. *russelii* venoms with the anti-venoms. (A) SDS-PAGE protein banding pattern of *Hh*v, *Ec*v, and *Dr*v, and M represents the molecular weight protein markers in kDa. (B) Western blots showing reactivity/cross-reactivity of *Hh*AV (Ba), *Ec*AV (Bb), *Dr*AV (Bc), BhAV (Bd), ViAV (Be), and pre-immune rabbit serum (Bf) with *Hh*v, *Ec*v, and *Dr*v. BSA, 25 μg was used as a negative control. (C) Corresponding Ponceau-S stain images showing the protein bands in respective membranes when stained before processing with respective anti-venoms in Western blot. In all cases, the electrophoresis was carried out under identical, non-reduced condition in 10% gels. (D) Indirect ELISA titers of *Hh*v, *Ec*v, and *Dr*v with anti-venoms. (Da) *Hh*v, (Db) *Ec*v, and (Dc) *Dr*v. Venoms, 0.1 μg/100 μl were independently treated with various dilutions of *Hh*AV, *Ec*AV, *Dr*AV, BhAV, and ViAV. The data is presented as Mean ± SEM (n = 4). (Antiserums, 1 mg/ml were adjusted, and used for dilution in ELISA).

The affinity-purified rabbit monovalent anti-venoms *Hh*AV, *Ec*AV, and *Dr*AV were prepared in the laboratory against *H*. *hypnale*, *E*. *carinatus*, and *D*. *russelii* venoms respectively. The equine polyvalent therapeutic anti-venoms BhAV and ViAV were made against the venoms of the ‘big four’ species, and are marketed in India. The above-mentioned monovalent and polyvalent anti-venoms were tested for their reactivity/cross-reactivity against the same three venoms. In Western blot studies, the monovalent anti-venoms *Hh*AV, *Ec*AV, and *Dr*AV readily reacted with their respective venoms (Figs 7B and S6). The reactivity was seen for the whole spectrum of the protein bands resolved in the respective gels. *Hh*AV rigorously reacted with its low molecular mass protein bands as suggested by intense bands compared to *Ec*AV and *Dr*AV which showed only marginal intensity (Fig 7Ba). Each anti-venom showed a marginal or insignificant cross-reactivity/paraspecific reactivity with the other two venoms. Interestingly, *Hh*AV did not show any signs of cross-reactivity with the *D*. *russelii* venom, while it showed marginal cross-reactivity with the *E*. *carinatus* venom where less conspicuous, cross-reacting bands appeared in the medium and low molecular mass range (Fig 7Bb and 7Bc). *Ec*AV did not show any signs of cross-reactivity with the *H*. *hypnale* venom while it showed signs of feeble cross-reactivity with the high molecular mass range protein bands of *D*. *russelii* venom (Fig 7Bb). In contrast, *Dr*AV showed significant cross-reactivity with *E*. *carinatus* venom while it showed marginal cross-reactivity with *H*. *hypnale* venom (Fig 7Bc). As expected, both BhAV and ViAV showed a significant degree of reactivity/cross-reactivity with both *E*. *carinatus* (Fig 7Bd) and *D*. *russelii* (Fig 7Be) venoms as suggested by intense reacting protein bands. Pre-immune rabbit serum showed insignificant/no cross-reactivity against *H*. *hypnale*, *E*. *carinatus*, and *D*. *russelii* venoms (Fig 7Bf). The anti-venoms readily reacted with high molecular mass protein bands while insignificant reactivity was seen with the medium and low molecular mass protein bands. Both BhAV and ViAV showed marginal cross-reactivity with *H*. *hypnale* venom, where the cross-reactivity was seen only with the high molecular mass protein bands. However, overall, ViAV revealed intense reactivity/cross-reactivity bands over BhAV with both *E*. *carinatus* and *D*. *russelii* venoms. In order to ensure the amount of venoms used for electrophoresis and eventual transfer on to PVDF membrane for Western blotting, the membranes were subjected to Ponceau-S stain before blocking for further analysis (Fig 7C). In order to quantify the extent of reactivity/cross-reactivity, indirect ELISA was performed. The wells were independently coated with an equal amount of *H*. *hypnale*, *E*. *carinatus*, and *D*. *russelii* venoms and incubated with various dilutions of anti-venoms (*Hh*AV/*Ec*AV/*Dr*AV/BhAV/ViAV). Respective secondary antibodies (Anti-rabbit or anti-equine) conjugated with HRP were used against primary antibody and quantified by measuring the absorbance which directly correlated to the binding efficiency of anti-venoms. The quantified values (n = 4) were plotted against the dilution factors of anti-venoms. The results revealed that *Hh*AV readily reacted with *H*. *hypnale* venom with a titer value of 1:78125, while *Ec*AV, *Dr*AV, BhAV, and ViAV failed to recognize even at a titer value of 1:5 (Fig 7Da). As expected, *Ec*AV, BhAV, and ViAV exhibited binding efficiency against the *E*. *carinatus* venom with a titer value of 1:953125, 1:78125, and 1:390625 respectively (Fig 7Db). However, *Dr*AV revealed the reactivity with its venom with a titer value of 1:390625 while BhAV and ViAV reacted/cross-reacted with a titer value of 1:78125 (Fig 7Dc). Thus, among *Hh*AV, *Ec*AV, and *Dr*AV monovalent anti-venoms, *Ec*AV exhibited comparatively a high titer value over the other two, and thus the reacting ability with their respective venoms varied as *Ec*AV >*Hh*AV >*Dr*AV. In our study based on the titer values, the antigenicity/immunogenicity of these three snake venoms varied as *E*. *carinatus* venom > *H*. *hypnale* venom > *D*. *russelii* venom. However, the varied titer values appear complex and may vary between the species or within the species of animals used for raising antibody. Further, the adjuvants used, and the emulsification time may also influence the antibody titer values. Subtle differences between the observed titer values in indirect ELISA and the intensity of bands in Western blots may be due to non-specific binding in respective cases. Thus, understanding the finer details of antigenicity/immunogenicity of snake venoms is highly challenging.

### Neutralization of *H*. *hypnale*, *E*. *carinatus*, and *D*. *russelii* venoms by monovalent and therapeutic polyvalent anti-venoms

**Fig 8:**
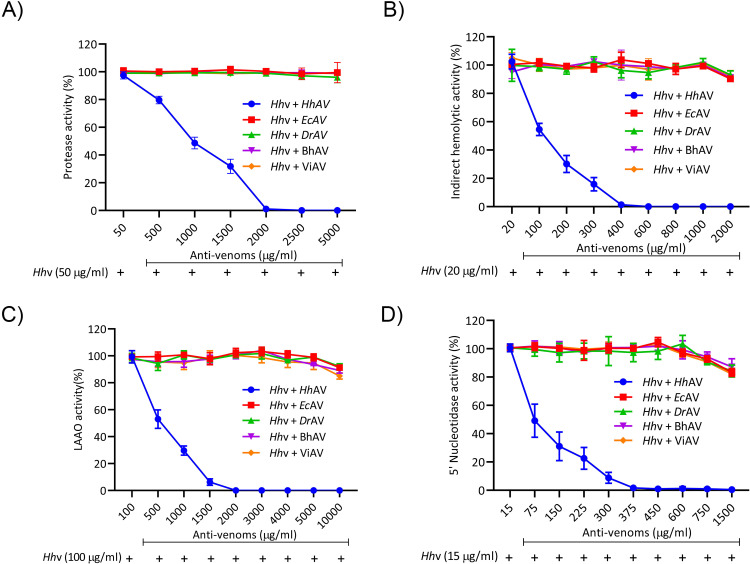
Neutralization of biochemical activities of *H*. *hypnale* venom by anti-venoms. (A) Proteolytic, (B) Indirect hemolytic, (C) L-Amino acid oxidase, and (D) 5’-Nucleotidase activities. *Hh*v was independently pre-incubated with various doses (50–10000 μg/ml) of *Hh*AV, *Ec*AV, *Dr*AV, BhAV, and ViAV for 15 min at room temperature. Protease activity; 50 μg/ml of *Hh*v alone was considered as 100% activity, hemolytic activity; 20 μg/ml of *Hh*v alone was considered as 100% activity, L-amino acid oxidase activity; 100 μg/ml of *Hh*v alone was considered as 100% activity, and 5’-Nucleotidase activity; 15 μg/ml of *Hh*v alone was considered as 100% activity. The data is presented as Mean ± SEM (n = 3).

**Fig 9:**
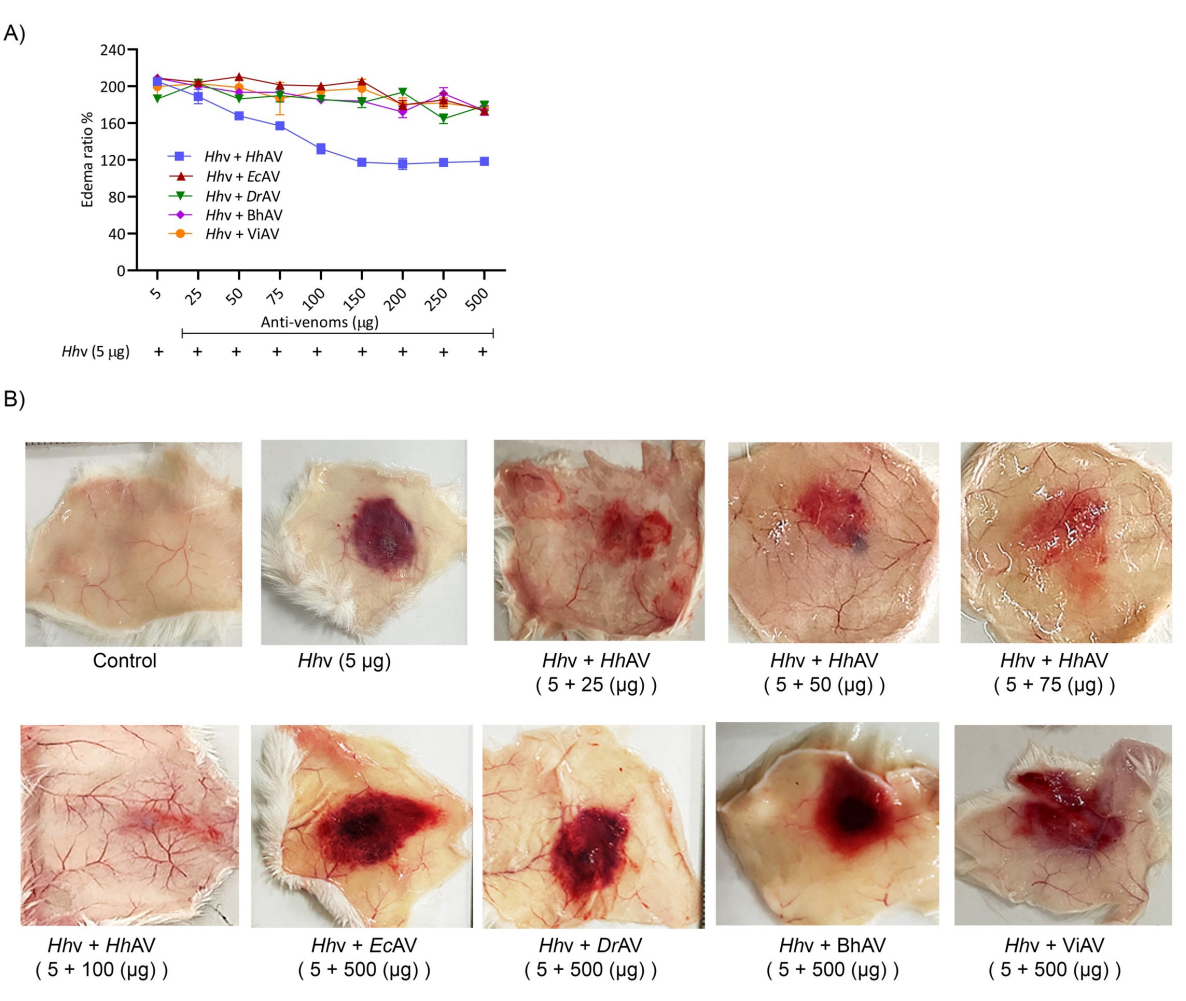
Neutralization of edema inducing activity and hemorrhagic activity of *H*. *hypnale* venom by anti-venoms using murine model. (A) Edema inducing activity, and (B) hemorrhagic activity of *Hh*v; in both cases, *Hh*v was independently pre-incubated with various doses (50–500 μg) of *Hh*AV, *Ec*AV, *Dr*AV, BhAV, and ViAV for 15 min at room temperature. *Hh*v, 5 μg alone was considered as 200% edema-inducing activity, and 5 μg *Hh*v alone was considered as 100% hemorrhagic activity. The data is presented as Mean ± SEM (n = 3).

**Fig 10:**
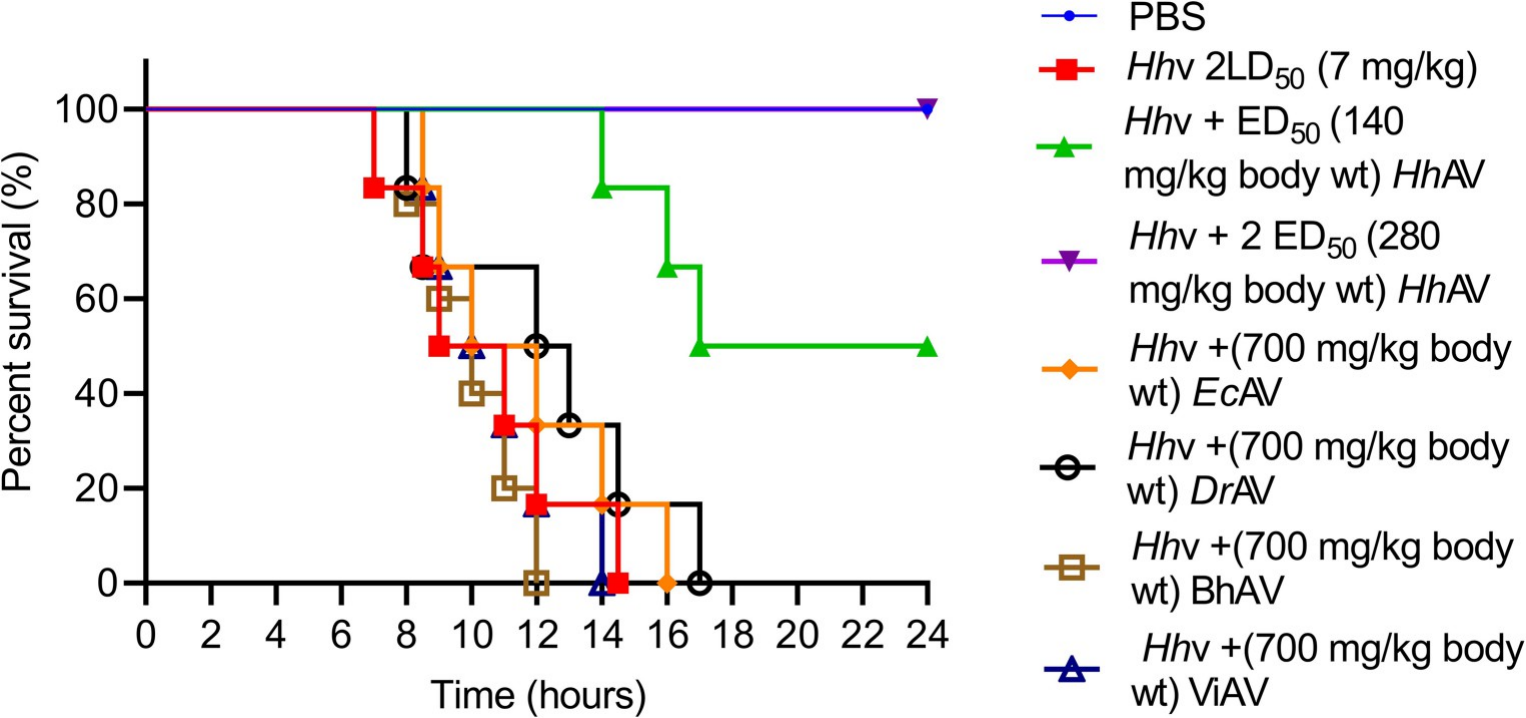
Neutralization of *H*. *hypnale* venom lethality by anti-venoms using the murine model. Groups (n = 6) of mice were independently injected intraperitoneally (i.p.) with 2 LD_50_ (7 mg/kg) dose of *Hh*v in 50 μl of PBS. Post 10 min of venom injection, the mice were administered with ED_50_ (140 mg/kg), and 2 ED_50_ (280 mg/kg) doses of *Hh*AV, and 700 mg/kg body weight of *Ec*AV, *Dr*AV, BhAV, and ViAV independently via tail vein (i.v.). Groups of mice that received venom alone and PBS were served as control experiments. Mice were kept under observation for 24 h and the time of death was recorded. The percent survival analysis of mice was done by constructing the Kaplan-Meier survival curve, the *p*-value was calculated using the log-rank (Mantel-Cox) test, ***p < 0.001, and **** *p* < 0.0001.

**Fig 11:**
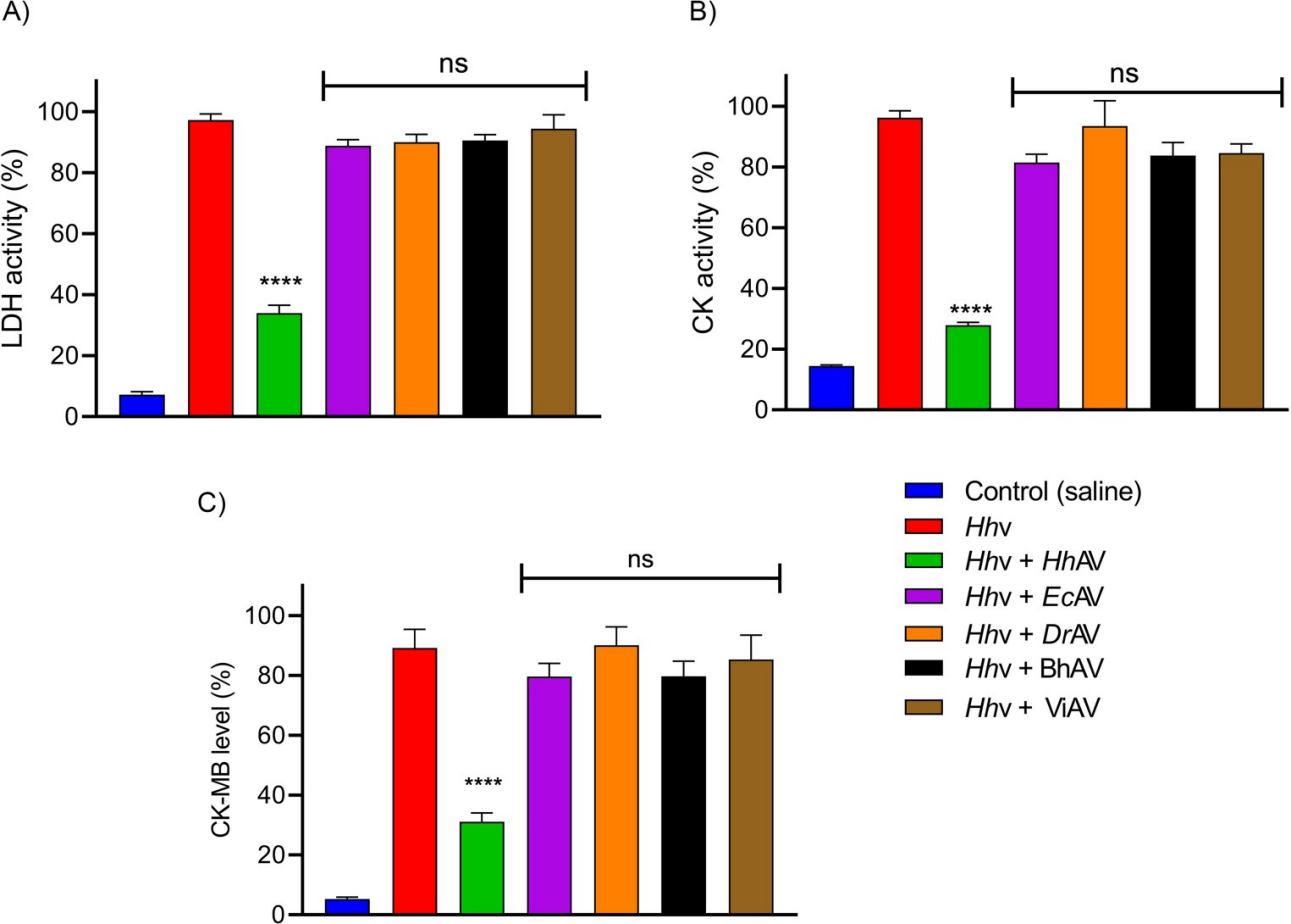
Neutralization of *H*. *hypnale* venom-induced myotoxicity and cardiotoxicity by anti-venoms using the murine model. Groups (n = 4) of mice were independently injected intramuscularly (i.m.) with LD_50_ dose (3.5 mg/kg) of *Hh*v in 50 μl of PBS. Post 10 min of venom injection, the mice were administered with ED_50_ (140 mg/kg) dose of *Hh*AV and 700 mg/kg doses of *Ec*AV, *Dr*AV, BhAV, and ViAV independently via tail vein (i.v.). The groups of mice that received PBS alone were served as control experiments. After 24 h, mice were anesthetized using xylazine; blood was drawn by cardiac puncture. The marker enzymes, LDH (A), CK (B), and CK-MB (C) activities (Units/L) were assayed in serum. The data is presented as Mean ± SEM (n = 4) and analyzed using one-way ANOVA followed by Bonferroni post-tests, ‘**** *p* <0.0001, and ns (not significant) *p* >0.05. ‘*’ significant compared to *Hh*v + *Hh*AV.

The affinity-purified rabbit monovalent anti-venoms (*Ec*AV and *Dr*AV) and equine polyvalent therapeutic anti-venoms (BhAV and ViAV) failed to neutralize the *H*. *hypnale* venom, while *Hh*AV neutralized all the properties of its venom efficiently. *Ec*AV, *Dr*AV, BhAV, and ViAV did not neutralize the biochemical properties such as proteolytic, indirect hemolytic, L-amino acid oxidase, and 5’-nucleotidase activities of *H*. *hypnale* venom even at an anti-venom dose of 10000 μg/ml (Fig 8). In contrast, *Hh*AV neutralized the activities of its venom efficiently at a dose of 2000 μg/ml. The indirect hemolytic and 5’-nucleotidase activities were neutralized efficiently at an *Hh*AV dose of <500 μg/ml (95% confidence limits: 80.45–373.83 μg/ml) (Fig 8B and 8D), while proteolytic, and LAAO activities were neutralized at a higher dose of 2000 μg/ml (95% CL: 454.11–1974.45 μg/ml) (Fig 8A and 8C). Further, *Hh*AV efficiently neutralized the edema-inducing and hemorrhagic activities of its venom at a dose <200 μg (95% CL: 37.67–157.32 μg) (Fig 9), whereas the coagulant activities such as plasma re-calcification time, APTT, PT, and TCT were neutralized at a dose of <600 μg/ml (95% CL: 88.58–602.84 μg/ml) (Table 2). *Ec*AV, *Dr*AV, BhAV, and ViAV did not neutralize the pathological properties such as edema-inducing, hemorrhagic and coagulant activities, myotoxicity, and cardiotoxicity of *H*. *hypnale* venom. As expected, *Ec*AV, *Dr*AV, BhAV, and ViAV showed nearly similar neutralization potencies against the biochemical and pathological properties and lethal toxicity of *E*. *carinatus* and *D*. *russelii* venoms. *Ec*AV, BhAV, and ViAV neutralized the proteolytic and LAAO activities of *E*. *carinatus* venom at a dose of <3000 μg/ml (95% CL: 384.17–2078.75 μg/ml), whereas the indirect hemolytic and 5’-nucleotidase activities were neutralized at a dose of <800 μg/ml (95% CL: 63.17–736.67 μg/ml) (S7 Fig). However, edema-inducing and hemorrhagic activities were neutralized much efficiently at a lesser dose of <250 μg (95% CL: 24.55–212.13 μg) (S9A and S9C Fig). Similarly, *Dr*AV, BhAV, and ViAV neutralized the proteolytic and LAAO activities of *D*. *russelii* venom at a dose of <3000 μg/ml (95% CL: 425.28–2492.21 μg/ml) while the indirect hemolytic and 5’-nucleotidase activities were neutralized at a dose <800 μg/ml (95% CL: 62.5–737.44 μg/ml) (S8 Fig). However, the edema-inducing and hemorrhagic activities were neutralized effectively at a much lower dose of <300 μg (95% CL: 12.09–249.22 μg) (S9B and S9D Fig). Thus, wherever neutralization was achieved, the anti-venoms, *Hh*AV/*Ec*AV/*Dr*AV/BhAV/ViAV neutralized the biochemical and pathological properties of *H*. *hypnale*, *E*. *carinatus*, and *D*. *russelii* venoms at a venom to anti-venom ratio of 1: 30–60 (w/w). Finally, it was critical to understand the neutralization efficacy of anti-venoms (monovalent/polyvalent) as it was much needed to neutralize the lethal toxicity of venoms in order to save the experimental mice. The groups of mice (n = 6) were independently injected intraperitoneally (i.p.) with the 2LD_50_ dose of *H*. *hypnale*/*E*. *carinatus*/*D*. *russelii* venoms and 10 mins later, different doses of anti-venoms, *Hh*AV/*Ec*AV/*Dr*AV/BhAV/ViAV were administered intravenously (i.v.) and the Kaplan-Meier survival curve was constructed to determine the ED_50_ dose of anti-venoms. With an ED_50_ dose of 140 mg/kg (95% CL: 52.7–308.95 mg/kg) weight, the *Hh*AV efficiently protected the mice from death while *Ec*AV, *Dr*AV, BhAV, and ViAV did not protect the mice even at the dose of 700 mg/kg weight (Fig 10 and S10) against *H*. *hypnale* venom lethality (Increments of anti-venom, ED_50_ was administered at 1 h intervals to reach 700 mg/kg weight). Similarly, *Ec*AV revealed an ED_50_ dose of 75 mg/kg (95% CL: 31.09–185.57 mg/kg) weight against *E*. *carinatus* venom lethality, while *Dr*AV revealed an ED_50_ dose of 90 mg/kg (95% CL: 41.4–258.6 mg/kg) weight against *D*. *russelii* venom lethality. In contrast, BhAV and ViAV showed the ED_50_ doses of 200 mg/kg (95% CL: 63.17–336.82 mg/kg) weight and 150 mg/kg (95% CL: 48.02–280.55 mg/kg) weight against *E*. *carinatus* venom lethality respectively. Similarly, BhAV and ViAV showed the ED_50_ doses of 210 mg/kg (95% CL: 81.59–328.41 mg/kg) weight and 180 mg/kg (95% CL: 57.7–372.31 mg/kg) weight against *D*. *russelii* venom lethality respectively (S11 Fig). Thus, among the monovalent anti-venoms, *Hh*AV showed comparatively less neutralizing efficacy over *Ec*AV and *Dr*AV for neutralizing the lethal potency of their respective venoms. Further, between BhAV and ViAV, the latter showed better neutralizing efficacy against the lethal potency of *E*. *carinatus* and *D*. *russelii* venoms. Additionally, the myotoxicity and cardiotoxicity induced by the LD_50_ dose (3.5 mg/kg) of *H*. *hypnale* venom were effectively neutralized by the ED_50_ (140 mg/kg) dose of *Hh*AV (Fig 11). Several authors have reported the clinical ineffectiveness of both BhAV and ViAV polyvalent anti-venoms against *H*. *hypnale* envenoming [5, 39, 83]. However, the monovalent anti-venom prepared against the Malayan pit viper *Calloselasma rhodostoma* and the Thailand therapeutic hemato polyvalent anti-venom were both found to neutralize the hemorrhagic, procoagulant, and necrotic activities, and the lethality of *H*. *hypnale* venom in a rodent model [9, 46, 47, 83–86]. Snake venoms are evolved to affect similar physiological targets, hemostatic or neuro-muscular, or both to immobilize, kill, and digest the prey animal. Thus, different snake venoms attack similar targets, maybe with varying degrees of affinity and specificity, thus functionally closely related. Generally, shape complementarity is the hallmark of interacting agents, viz. casein degrading property of proteases, phospholipid degrading property of PLA_2_s, platelet receptor binding property of disintegrins, and many more. Hence, similar toxins (enzymatic or non-enzymatic) from different snake venoms are expected to share some degree of similarity, at least in their interacting/binding sites, and therefore in their antigenicity as well. So, paradoxically, marginal or lack of formation of cross-reacting antibodies underscore the immunological distinctness of homologous snake venoms. Thus, understanding the immunological uniqueness of these venoms/toxins appears highly interesting and complex and therefore puts forward an exciting academic challenge.

In summary, as *H*. *hypnale* venom is not marketed in India, this offers a serious setback for its detailed characterization. Besides, the *H*. *hypnale* bite has persisted with no anti-venom therapy in India. Thus, this study has made a sincere attempt to systematically compare the biochemical, pathological, and immunological properties of Sri Lankan *H*. *hypnale* venom with the venoms of Indian *E*. *carinatus*, and *D*. *russelii* snakes. The study has critically exposed the inadequate paraspecific neutralizing ability of Indian therapeutic equine polyvalent (BhAV and ViAV), and rabbit monovalent (*Ec*AV and *Dr*AV) anti-venoms against *H*. *hypnale* venom lethal toxicity. However, considering one of the key limitations, the lack of information on the offended snake in most cases, it is appropriate to use the polyvalent anti-venom for the treatment purpose. Incorporating *H*. *hypnale* venom into the anti-venom production regimen in the Western Ghats region would be a great relief. Therefore, it is time for India to review the `big four‘ concept based on the geography. The Western Ghats of India and Sri Lanka being the epicenters of *H*. *hypnale* envenoming, it is important to explore the possible variability in venom composition in the regions. Further, it is worth praising the ongoing efforts of the University of Peradeniya in Sri Lanka. They have a successful network with Animal Venom Research International (AVRI) to produce appropriate polyspecific neutralizing anti-venoms to treat snake envenoming, including the *H*. *hypnale* bite [87,88]. Regrettably, except for cursing their ill fate, the Indian victims have no choice, but to live with the dreadful effects of *H*. *hypnale* bite.

**Table 2:** Neutralization of coagulant activity (Plasma re-calcification time, APTT, and PT assays), and thrombin-like activity of *H*. *hypnale* venom by anti-venoms: *Hh*v was independently pre-incubated with various doses (100–2000 μg/ml) of *Hh*AV, *Ec*AV, *Dr*AV, BhAV, and ViAV for 15 min at room temperature. In all cases, 20 μg/ml of *Hh*v alone was considered as 100% coagulant activity and thrombin-like activity. The data is presented as Mean ± SEM (n = 4) and analyzed using one-way ANOVA followed by Bonferroni post-tests, ‘**** *p* <0.0001, *** *p* <0.001, ** *p* <0.01, * *p* <0.05, and ns (not significant) *p* >0.05. ‘*’ significant compared to the control group (PBS). ‘*’ significant compared to *Hh*v+ *Hh*AV.

Group	Venom + anti-venom (μg/ml)	Plasma re-calcification (Time in sec)	APTT assay (Time in sec)	PT assay (Time in sec)	Thrombin clotting time (Time in sec)
PBS (Control)	-	319 ± 6	38 ± 3	22 ± 1	≥600
*Hh*v	20 + 0	92 ± 2	8 ± 1	13 ± 1	179 ± 5
*Hh*v *+ Hh*AV	20 + 600****	325 ± 2	39 ± 2	23 ± 2	≥ 600
*Hh*v *+ Ec*AV	20 + 2000^ns^	84 ± 1	9 ± 2	13 ± 2	253 ± 10
*Hh*v *+ Dr*AV	20 + 2000^ns^	88 ± 2	7 ± 1	10 ± 1	184 ± 15
*Hh*v *+* BhAV	20 + 2000^ns^	85 ± 1	8 ± 2	14 ± 2	169 ± 10
*Hh*v+ ViAV	20 + 2000^ns^	94 ± 1	9 ± 2	13 ± 1	172 ± 8

## Supporting information

S1 Fig SDS-PAGE banding patterns of *H. hypnale, E. carinatus*, and *D. russelii* venoms. (a) SDS-PAGE, *Hh*v, *Ec*v, and *Dr*v were analyzed by 10% SDS-PAGE under non-reduced condition. (b) Corresponding densitometric analysis (ImageJ Software Ver. 1.53k, USA) of the gel. (c) SDS-PAGE (10%) of *Dr*v, *Ec*v, and *Hh*v under both non-reduced and reduced conditions. (d) SDS-PAGE (12.5%) of *Dr*v, *Ec*v, and *Hh*v under both non-reduced and reduced conditions. In all cases, 25 μg each of venom were loaded, and M represents the molecular weight markers. The gels were stained and visualized by 0.25% of Coomassie Brilliant Blue (R-250) staining. After destaining, the images were captured by HP Scanjet (Model-G2410).
(TIF)

S2 Fig Caseinolytic and gelatinolytic zymograms of *H. hypnale, E. carinatus*, and *D. russelii* venoms. (A) Caseinolytic activity and (B) gelatinolytic activity, different doses (5, 10, 20, and 30 μg) each of *Hh*v, *Ec*v, and *Dr*v were resolved in 10% SDS-PAGE under non-reduced condition. Casein and gelatin (0.2%) were incorporated as substrates into respective gels. In all cases, 20 μg of BSA was used as a negative control and 0.1 μg of trypsin was used as a positive control. The gels were stained with 0.25% of Coomassie Brilliant Blue (R-250) staining. After destaining, the images were captured by using HP Scanjet (Model-G2410). The clear translucent zones against a blue background indicate the caseinolytic and gelatinolytic activities of venoms in respective gels.
(TIF)

S3 Fig Hyaluronidase activity of *H. hypnale, E. carinatus*, and *D. russelii* venoms. (a) Hyaluronidase activity, 0.017% of hyaluronic acid was incorporated as a substrate into 10% SDS-PAGE and 50 μg each of *Hh*v, *Ec*v, and *Dr*v were analyzed under non-reduced condition. M. represents the molecular weight protein markers in kDa. (b) The corresponding densitometric (ImageJ Software Ver. 1.53k, USA) analysis of hyaluronidase activity of venoms.
(TIF)

S4 Fig Deoxyribonuclease (DNase) activity of *H. hypnale, E. carinatus*, and *D. russelii* venoms. Calf thymus DNA (2 kb), 250 ng was independently treated with the venoms (50 μg) for 60 min, at 37°C in a final volume of 30 μl PBS and analyzed in 1.2% agarose gel electrophoresis. Lane 1 DNA alone, lane 2 DNase 1 (10 units), lane 3 *Hh*v, lane 4 *Ec*v, and lane 5 *Dr*v were loaded. After electrophoresis, the gel was visualized and photographed on an ultraviolet transilluminator (Alliance 2.7, Uvitech).
(TIF)

S5 Fig Murine model of *H. hypnale, E. carinatus*, and *D. russelii* venoms induced tail tissue destruction. (A) Graph showing the semi-quantitative representation of tail tissue injury score of respective venoms. The groups (n = 3) of mice were independently injected subcutaneously with LD_50_ dose of each of the venom into the mice tail, 3 cm from the tip of the tail. (B) *Hh*v, (C) *Ec*v, and (D) *Dr*v respectively. Mice injected with 50 μl of PBS alone was served as a control experiment. The data is presented as Mean ± SEM (n = 3).
(TIF)

S6 Fig Reactivity/cross-reactivity of *H. hypnale, E. carinatus, D. russelii* and *N. naja* venoms with the anti-venoms. (A) Western blots showing reactivity/cross-reactivity of *Hh*AV (Aa), *Ec*AV (Ab), *Dr*AV (Ac), BhAV (Ad), and ViAV (Ae) with *Hh*v, *Ec*v, *Dr*v, and *Nn*v. (B) Corresponding PVDF membranes showing unedited/uncropped images of respective western blots.
(TIF)

S7 Fig Neutralization of biochemical activities of *E. carinatus* venom by anti-venoms. (A) Proteolytic activity, (B) Indirect hemolytic activity, (C) L-Amino acid oxidase activity, and (D) 5’-Nucleotidase activity. For the neutralization study, *Ec*v was independently pre-incubated with various amounts (100–3000 μg/ml) of anti-venoms (*Ec*AV/BhAV/ViAV) for 15 min at room temperature. Protease activity of 50 μg/ml of *Ec*v was considered as 100% activity. The indirect hemolytic activity caused by 20 μg/ml of *Ec*v was considered as 100% activity. LAAO due to 100 μg/ml of *Ec*v was considered as 100% activity. The 5’-Nucleotidase activity caused by 40 μg/ml of *Ec*v was considered as 100% activity. The data is presented as Mean ± SEM (n = 3).
(TIF)

S8 Fig Neutralization of biochemical activities of *D. russelii* venom by anti-venoms. (A) Proteolytic activity, (B) Indirect hemolytic activity, (C) L-Amino acid oxidase activity, and (D) 5’-Nucleotidase activity. For the neutralization study, *Dr*v was independently pre-incubated with various doses (100–3000 μg/ml) of anti-venoms (*DrAV*/BhAV/ViAV) for 15 min at room temperature. Protease activity of 100 μg/ml of *Dr*v was considered as 100% activity. The indirect hemolytic activity caused by 20 μg/ml of *Dr*v was considered as 100% activity. LAAO due to 100 μg/ml of *Dr*v was considered as 100% activity. The 5’-Nucleotidase activity caused by 30 μg/ml of *Dr*v was considered as 100% activity. The data is presented as Mean ± SEM (n = 3).
(TIF)

S9 Fig Neutralization of *E. carinatus and D. russelii venoms* induced-edema and hemorrhagic activities by anti-venoms using the murine model. (A) *Ec*v was independently pre-incubated with various doses (25–200 μg) of anti-venoms (*Ec*AV/BhAV/ViAV) for 15 min at room temperature, 5 μg of *Ec*v alone was considered as 200% edema inducing activity. (B) *Dr*v was independently pre-incubated with various doses of anti-venoms (*Dr*AV/BhAV/ViAV) for 15 min at room temperature, 10 μg of *Dr*v alone was considered as 200% edema-inducing activity. (C) *Ec*v was independently pre-incubated with various doses of anti-venoms (*Ec*AV/BhAV/ViAV) for 15 min at room temperature, 5 μg of venom alone was considered as 100% hemorrhagic activity. (D) *Dr*v was independently pre-incubated with various doses (50–300 μg) of anti-venoms (*Dr*AV/BhAV/ViAV) for 15 min at room temperature, 5 μg of *Dr*v alone was considered as 100% hemorrhagic activity. The data is presented as Mean ± SEM (n = 3).
(TIF)

S10 Fig Neutralization of the lethality of *H. hypnale* venom by *Hh*AV using the murine model. Groups (n = 6) of mice were independently injected intraperitoneally (i.p.) with 2 LD_50_ doses of *Hh*v in 50 μl PBS. Post 10 min venom injection, the mice were independently administered with 70, 140, 210, 280, and 350 mg/kg body weight of *Hh*AV via mice tail vein (i.v.). Mice were kept under observation for 24 h and the time of death was recorded. Groups of mice that received venom alone and PBS alone were served as control experiments. The percent survival analysis of mice was done by constructing the Kaplan-Meier survival curve, the p-value was calculated using the log-rank (Mantel-Cox) test, ***p < 0.001, and **** *p* < 0.0001.
(TIF)

S11 Fig Neutralization of the lethality of *E. carinatus* and *D. russelii venoms* by anti-venoms using murine model. (A) Groups of mice (n = 6) were independently injected intraperitoneally (i.p.) with 2 LD_50_ (5 mg/kg of body weight) dose of *Ec*v in 50 μl PBS. Post 10 min venom injection, the mice were independently administered with 50, 75, 150, 200, 300, and 400 mg/kg of *Ec*AV, BhAV, and ViAV respectively via mice tail vein (i.v). The effective dose (ED_50_) value of *Ec*AV 75 mg/kg, BhAV 200 mg/kg, and ViAV 150 mg/kg were determined. (B) Groups of mice (n = 6) were independently injected intraperitoneally (i.p.) with 2 LD_50_ (5 mg/kg of body weight) dose of *Dr*v in 50 μl PBS. Post 10 min venom injection, the mice were independently administered with 60, 90, 120, 180, 210, 360, and 420 mg/kg of *Dr*AV, BhAV, and ViAV respectively via mice tail vein (i.v.). The effective dose value of *Dr*AV 90 mg/kg, BhAV 240 mg/kg), and ViAV 180 mg/kg were determined. In all the cases, the groups of mice that received respective venoms alone and PBS alone were served as control experiments. Mice were kept under observation for 24 h and the time of death was recorded. The percent survival analysis of mice was done by constructing the Kaplan-Meier survival curve, the p-value was calculated using the log-rank (Mantel-Cox) test, ***p < 0.001, and **** *p* < 0.0001.
(TIF)
