# Supplementary figures and images for: A comparative cross-reactivity and paraspecific neutralization study on Hypnale hypnale, Echis carinatus, and Daboia russelii monovalent and therapeutic polyvalent anti-venoms
Source: PLoS Negl Trop Dis. 2022 Mar 28;16(3):e0010292. doi: 10.1371/journal.pntd.0010292 (PMC9037957; doi:10.1371/journal.pntd.0010292)

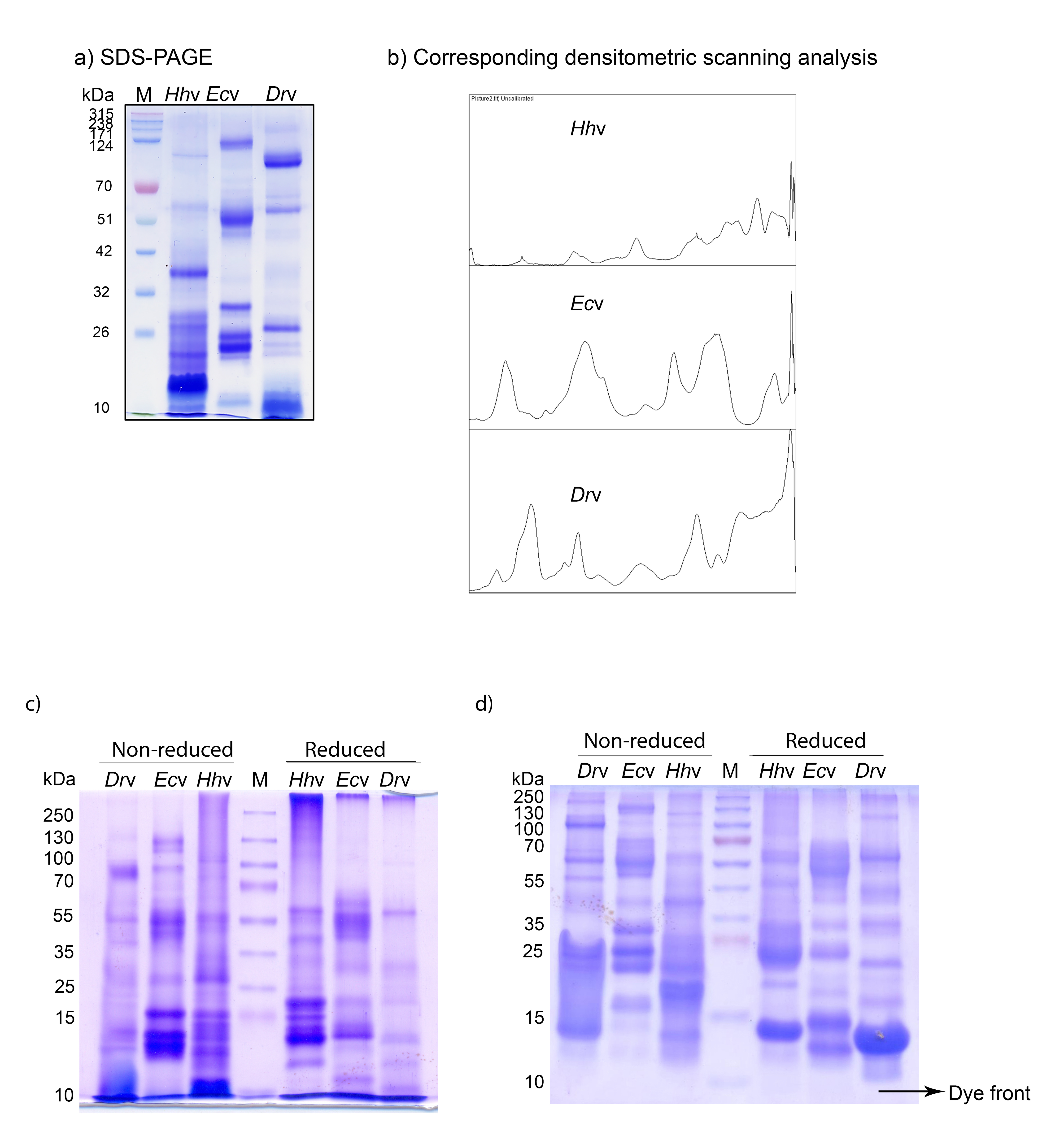

Supplement: S1 Fig — (a) SDS-PAGE, Hhv, Ecv, and Drv were analyzed by 10% SDS-PAGE under non-reduced condition. (b) Corresponding densitometric analysis (ImageJ Software Ver. 1.53k, USA) of the gel. (c) SDS-PAGE (10%) of Drv, Ecv, and Hhv under both non-reduced and reduced conditions. (d) SDS-PAGE (12.5%) of Drv, Ecv, and Hhv under both non-reduced and reduced conditions. In all cases, 25 μg each of venom were loaded, and M represents the molecular weight markers. The gels were stained and visualized by 0.25% of Coomassie Brilliant Blue (R-250) staining. After destaining, the images were captured by HP Scanjet (Model-G2410). (TIF) [file pntd.0010292.s001.tif]

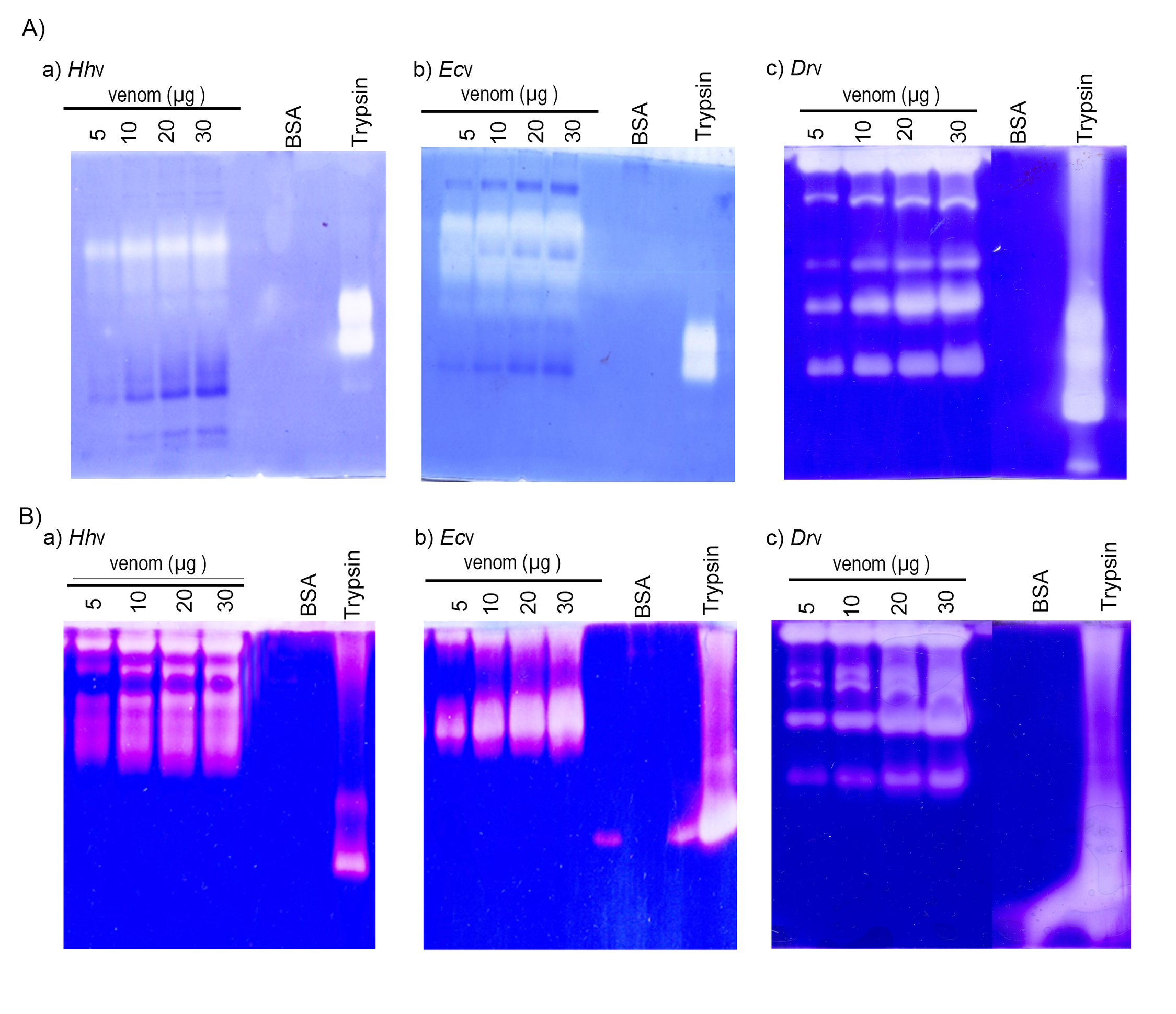

Supplement: S2 Fig — (A) Caseinolytic activity and (B) gelatinolytic activity, different doses (5, 10, 20, and 30 μg) each of Hhv, Ecv, and Drv were resolved in 10% SDS-PAGE under non-reduced condition. Casein and gelatin (0.2%) were incorporated as substrates into respective gels. In all cases, 20 μg of BSA was used as a negative control and 0.1 μg of trypsin was used as a positive control. The gels were stained with 0.25% of Coomassie Brilliant Blue (R-250) staining. After destaining, the images were captured by using HP Scanjet (Model-G2410). The clear translucent zones against a blue background indicate the caseinolytic and gelatinolytic activities of venoms in respective gels. (TIF) [file pntd.0010292.s002.tif]

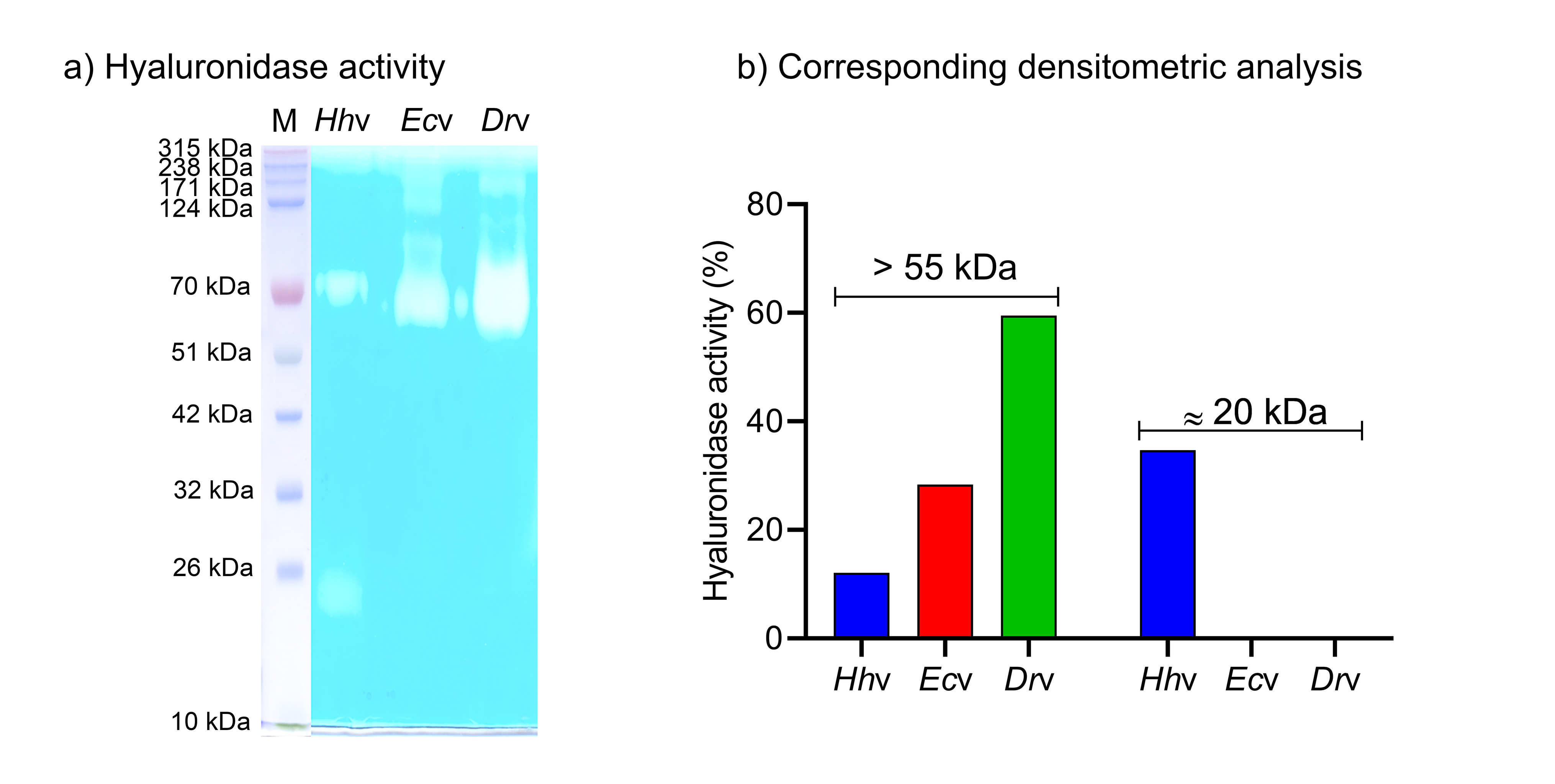

Supplement: S3 Fig — (a) Hyaluronidase activity, 0.017% of hyaluronic acid was incorporated as a substrate into 10% SDS-PAGE and 50 μg each of Hhv, Ecv, and Drv were analyzed under non-reduced condition. M. represents the molecular weight protein markers in kDa. (b) The corresponding densitometric (ImageJ Software Ver. 1.53k, USA) analysis of hyaluronidase activity of venoms. (TIF) [file pntd.0010292.s003.tif]

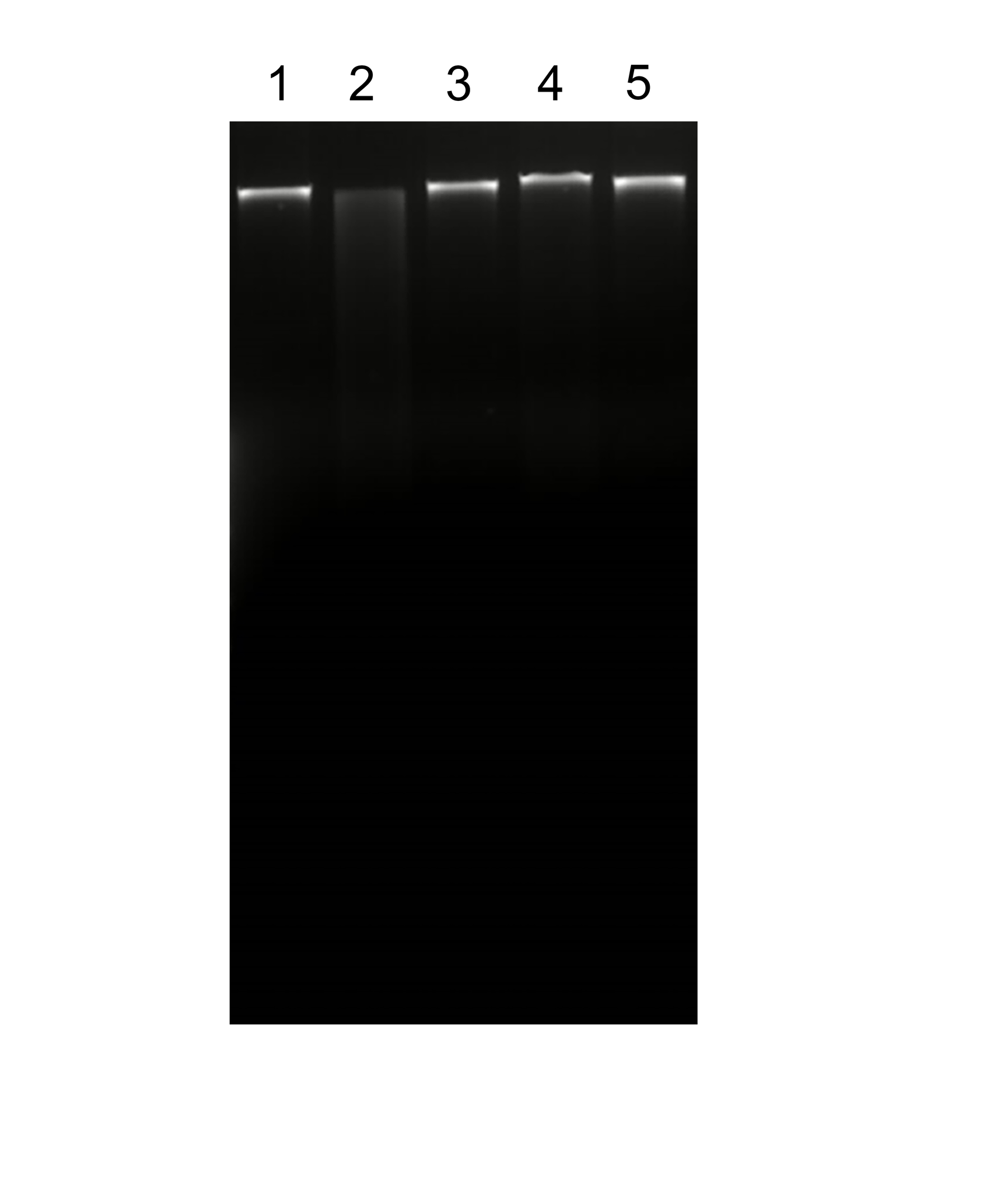

Supplement: S4 Fig — Calf thymus DNA (2 kb), 250 ng was independently treated with the venoms (50 μg) for 60 min, at 37°C in a final volume of 30 μl PBS and analyzed in 1.2% agarose gel electrophoresis. Lane 1 DNA alone, lane 2 DNase 1 (10 units), lane 3 Hhv, lane 4 Ecv, and lane 5 Drv were loaded. After electrophoresis, the gel was visualized and photographed on an ultraviolet transilluminator (Alliance 2.7, Uvitech). (TIF) [file pntd.0010292.s004.tif]

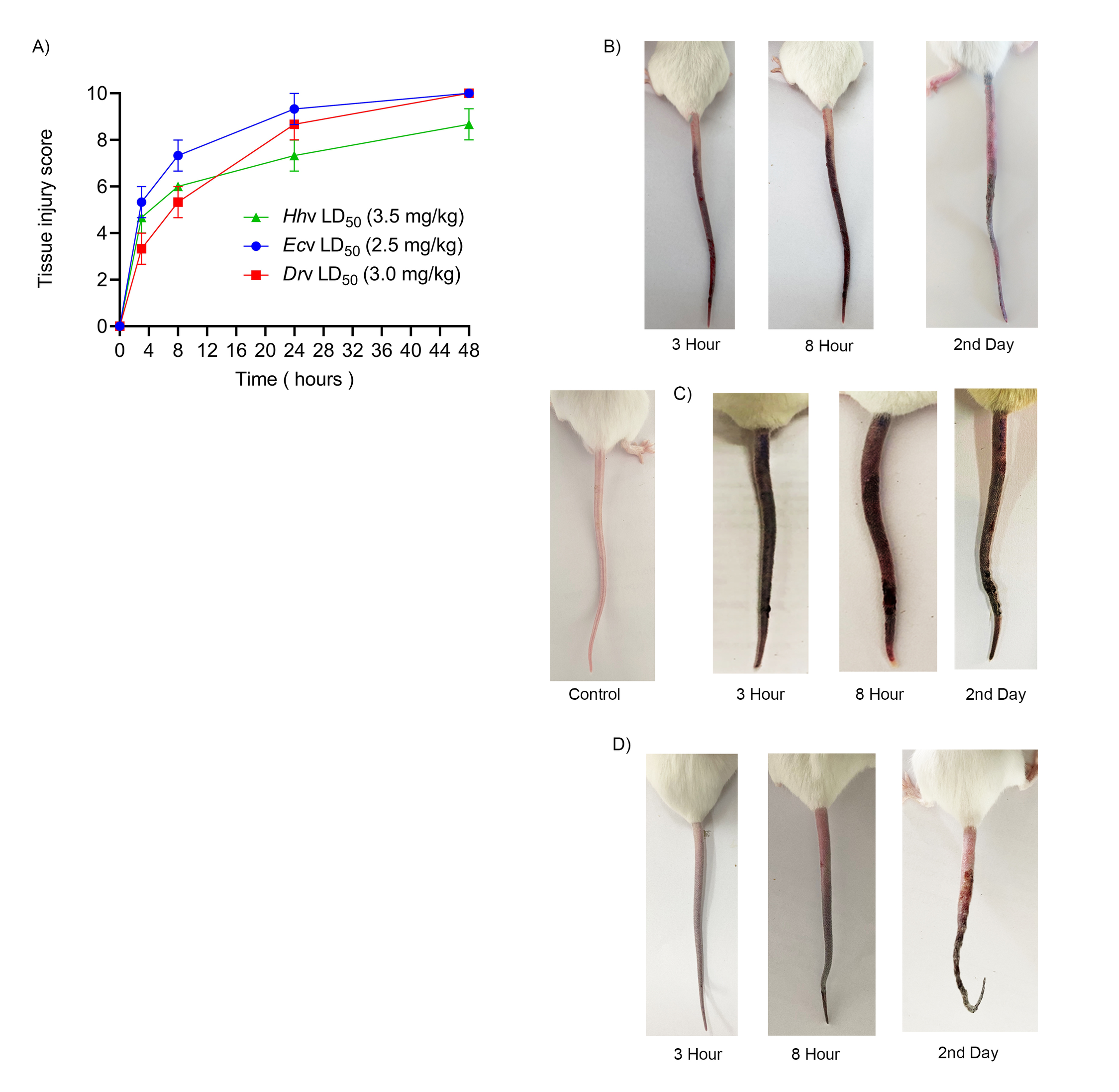

Supplement: S5 Fig — (A) Graph showing the semi-quantitative representation of tail tissue injury score of respective venoms. The groups (n = 3) of mice were independently injected subcutaneously with LD50 dose of each of the venom into the mice tail, 3 cm from the tip of the tail. (B) Hhv, (C) Ecv, and (D) Drv respectively. Mice injected with 50 μl of PBS alone was served as a control experiment. The data is presented as Mean ± SEM (n = 3). (TIF) [file pntd.0010292.s005.tif]

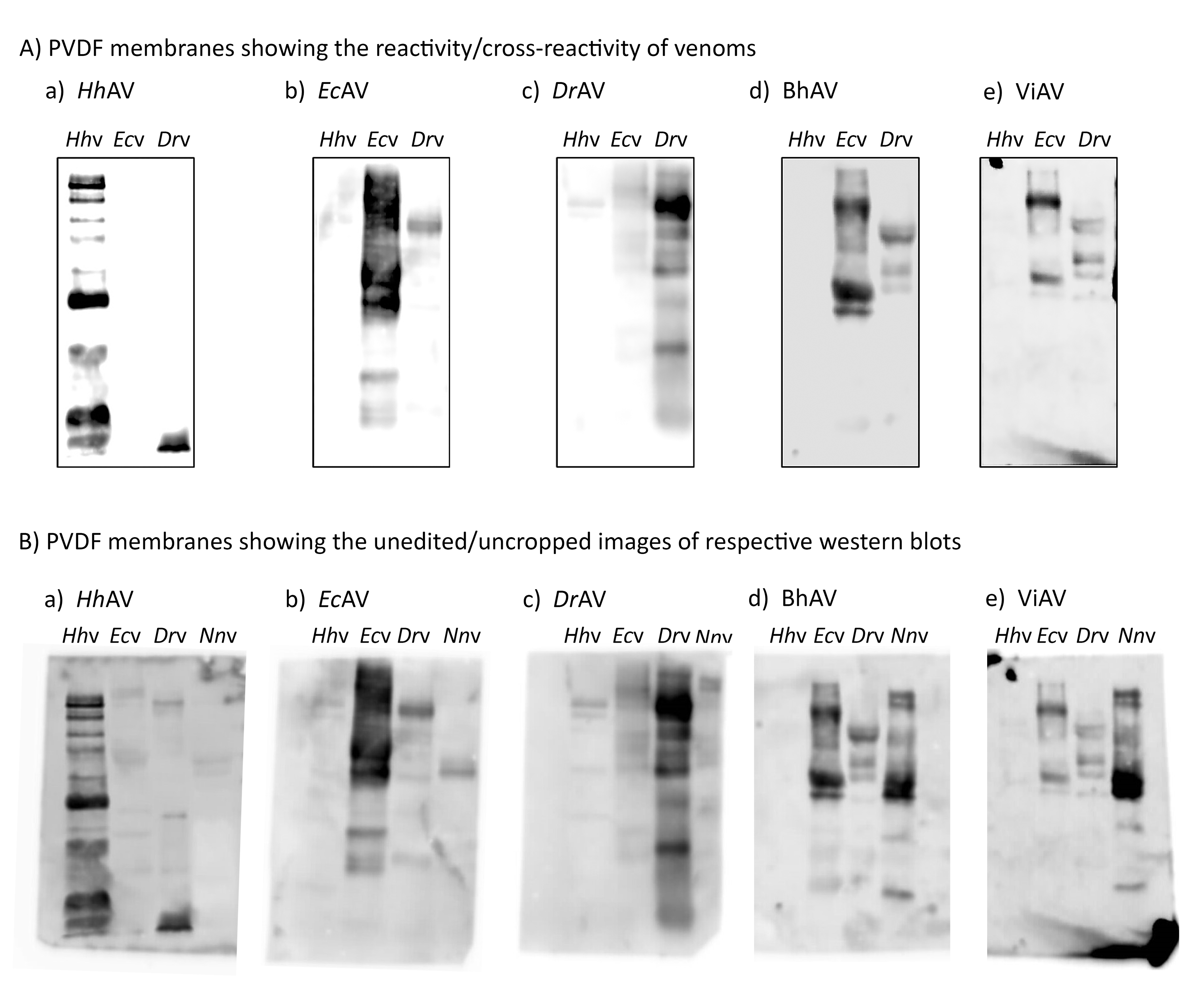

Supplement: S6 Fig — (A) Western blots showing reactivity/cross-reactivity of HhAV (Aa), EcAV (Ab), DrAV (Ac), BhAV (Ad), and ViAV (Ae) with Hhv, Ecv, Drv, and Nnv. (B) Corresponding PVDF membranes showing unedited/uncropped images of respective western blots. (TIF) [file pntd.0010292.s006.tif]

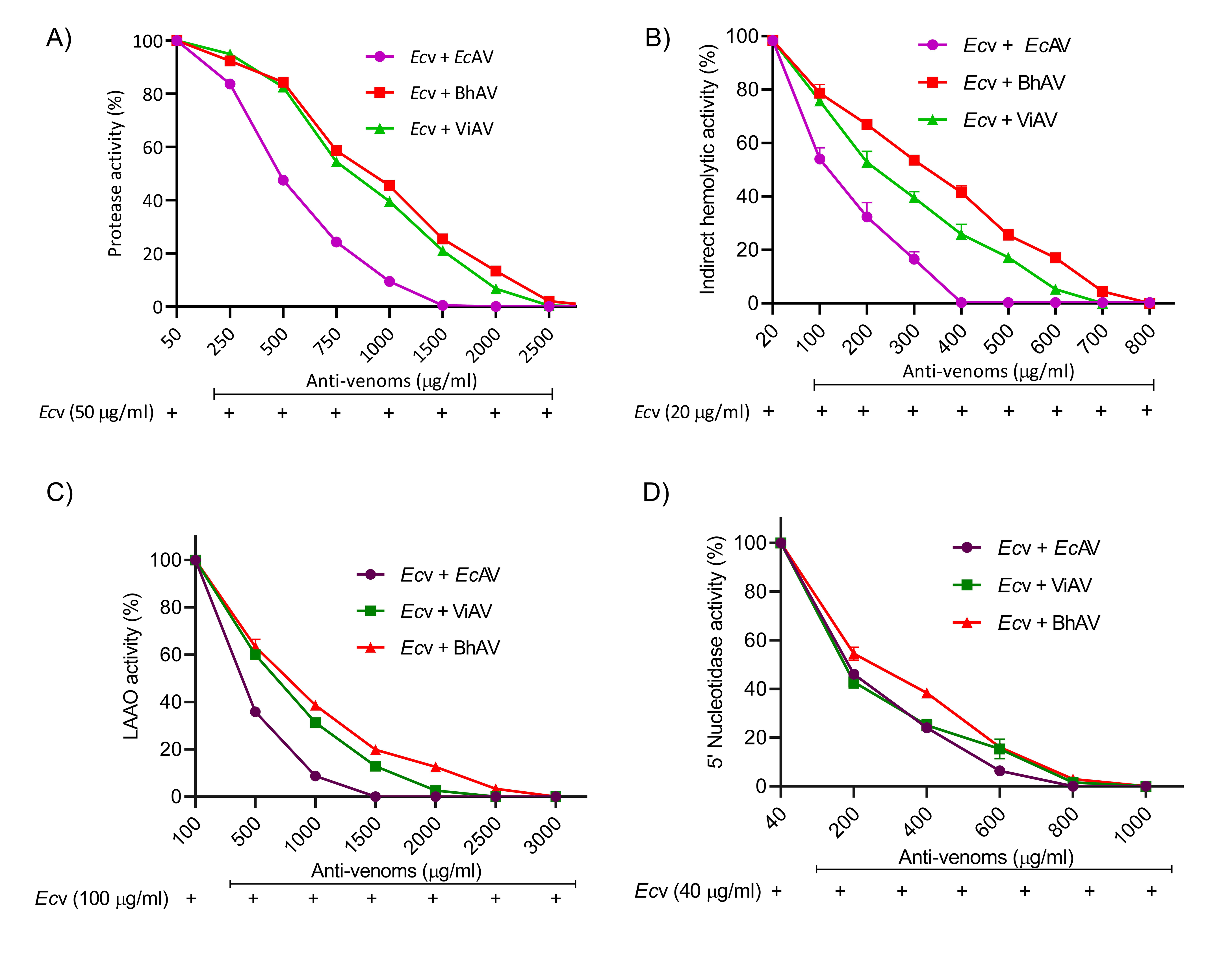

Supplement: S7 Fig — (A) Proteolytic activity, (B) Indirect hemolytic activity, (C) L-Amino acid oxidase activity, and (D) 5’-Nucleotidase activity. For the neutralization study, Ecv was independently pre-incubated with various amounts (100–3000 μg/ml) of anti-venoms (EcAV/BhAV/ViAV) for 15 min at room temperature. Protease activity of 50 μg/ml of Ecv was considered as 100% activity. The indirect hemolytic activity caused by 20 μg/ml of Ecv was considered as 100% activity. LAAO due to 100 μg/ml of Ecv was considered as 100% activity. The 5’-Nucleotidase activity caused by 40 μg/ml of Ecv was considered as 100% activity. The data is presented as Mean ± SEM (n = 3). (TIF) [file pntd.0010292.s007.tif]

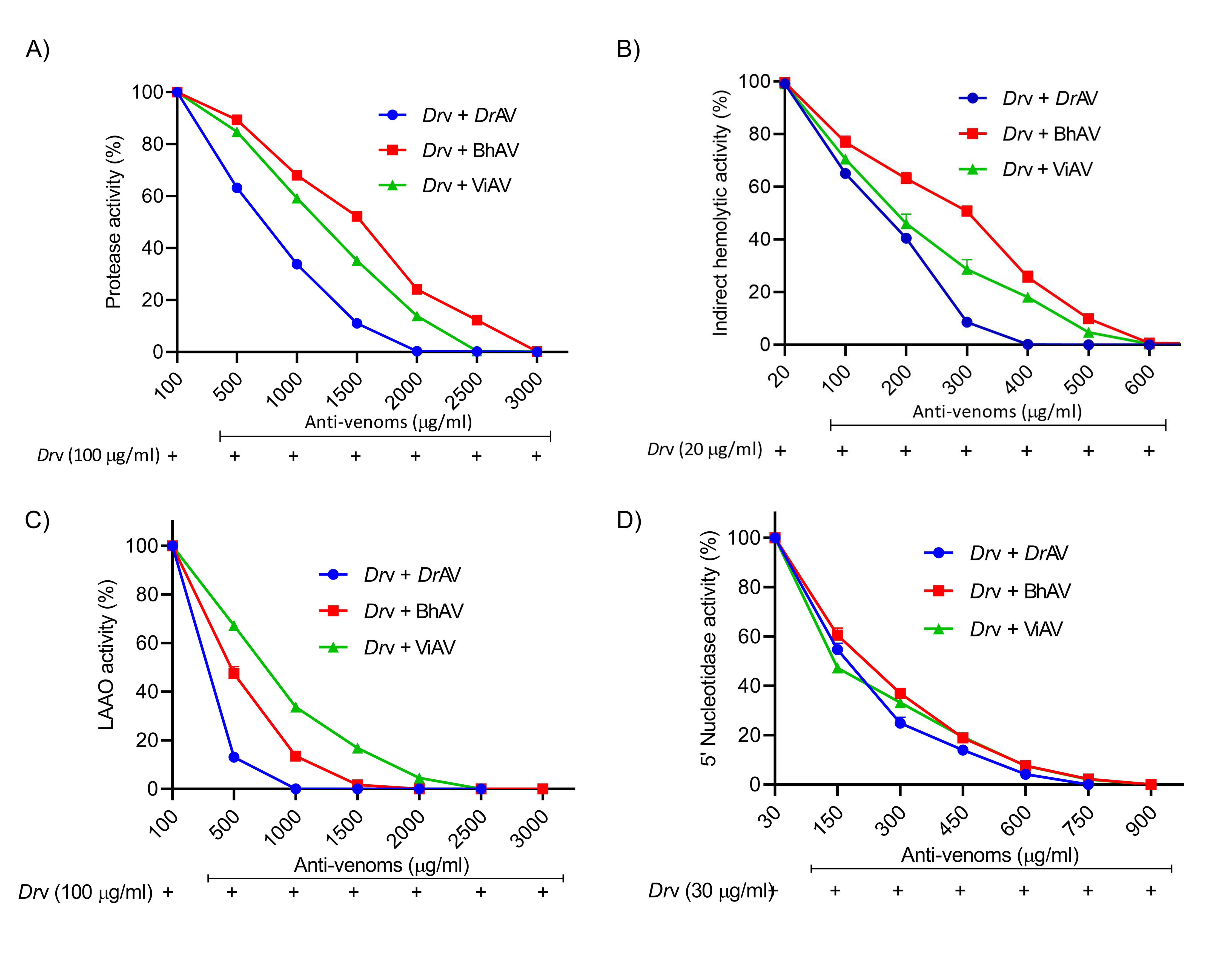

Supplement: S8 Fig — (A) Proteolytic activity, (B) Indirect hemolytic activity, (C) L-Amino acid oxidase activity, and (D) 5’-Nucleotidase activity. For the neutralization study, Drv was independently pre-incubated with various doses (100–3000 μg/ml) of anti-venoms (DrAV/BhAV/ViAV) for 15 min at room temperature. Protease activity of 100 μg/ml of Drv was considered as 100% activity. The indirect hemolytic activity caused by 20 μg/ml of Drv was considered as 100% activity. LAAO due to 100 μg/ml of Drv was considered as 100% activity. The 5’-Nucleotidase activity caused by 30 μg/ml of Drv was considered as 100% activity. The data is presented as Mean ± SEM (n = 3). (TIF) [file pntd.0010292.s008.tif]

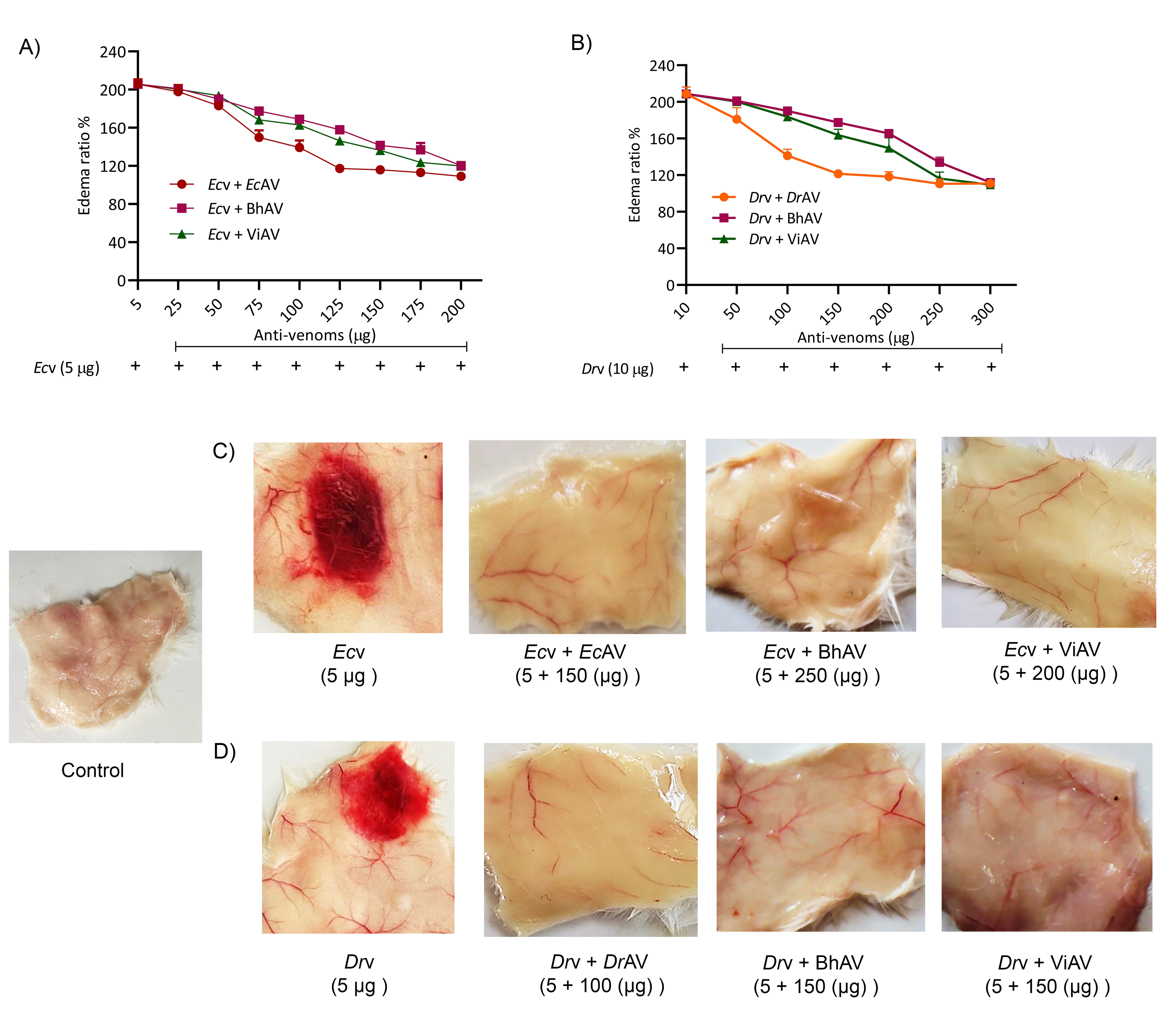

Supplement: S9 Fig — (A) Ecv was independently pre-incubated with various doses (25–200 μg) of anti-venoms (EcAV/BhAV/ViAV) for 15 min at room temperature, 5 μg of Ecv alone was considered as 200% edema inducing activity. (B) Drv was independently pre-incubated with various doses of anti-venoms (DrAV/BhAV/ViAV) for 15 min at room temperature, 10 μg of Drv alone was considered as 200% edema-inducing activity. (C) Ecv was independently pre-incubated with various doses of anti-venoms (EcAV/BhAV/ViAV) for 15 min at room temperature, 5 μg of venom alone was considered as 100% hemorrhagic activity. (D) Drv was independently pre-incubated with various doses (50–300 μg) of anti-venoms (DrAV/BhAV/ViAV) for 15 min at room temperature, 5 μg of Drv alone was considered as 100% hemorrhagic activity. The data is presented as Mean ± SEM (n = 3). (TIF) [file pntd.0010292.s009.tif]

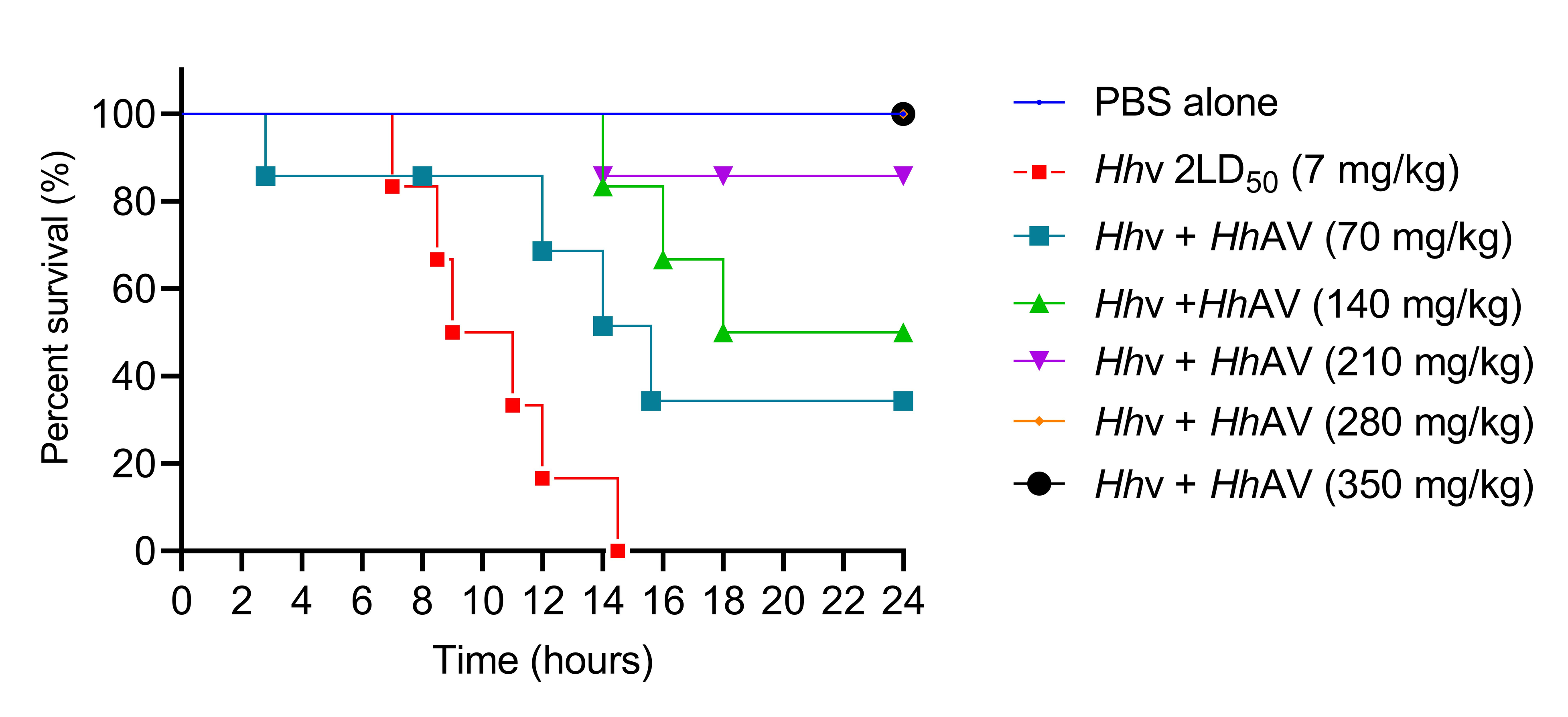

Supplement: S10 Fig — Groups (n = 6) of mice were independently injected intraperitoneally (i.p.) with 2 LD50 doses of Hhv in 50 μl PBS. Post 10 min venom injection, the mice were independently administered with 70, 140, 210, 280, and 350 mg/kg body weight of HhAV via mice tail vein (i.v.). Mice were kept under observation for 24 h and the time of death was recorded. Groups of mice that received venom alone and PBS alone were served as control experiments. The percent survival analysis of mice was done by constructing the Kaplan-Meier survival curve, the p-value was calculated using the log-rank (Mantel-Cox) test, ***p < 0.001, and **** p < 0.0001. (TIF) [file pntd.0010292.s010.tif]

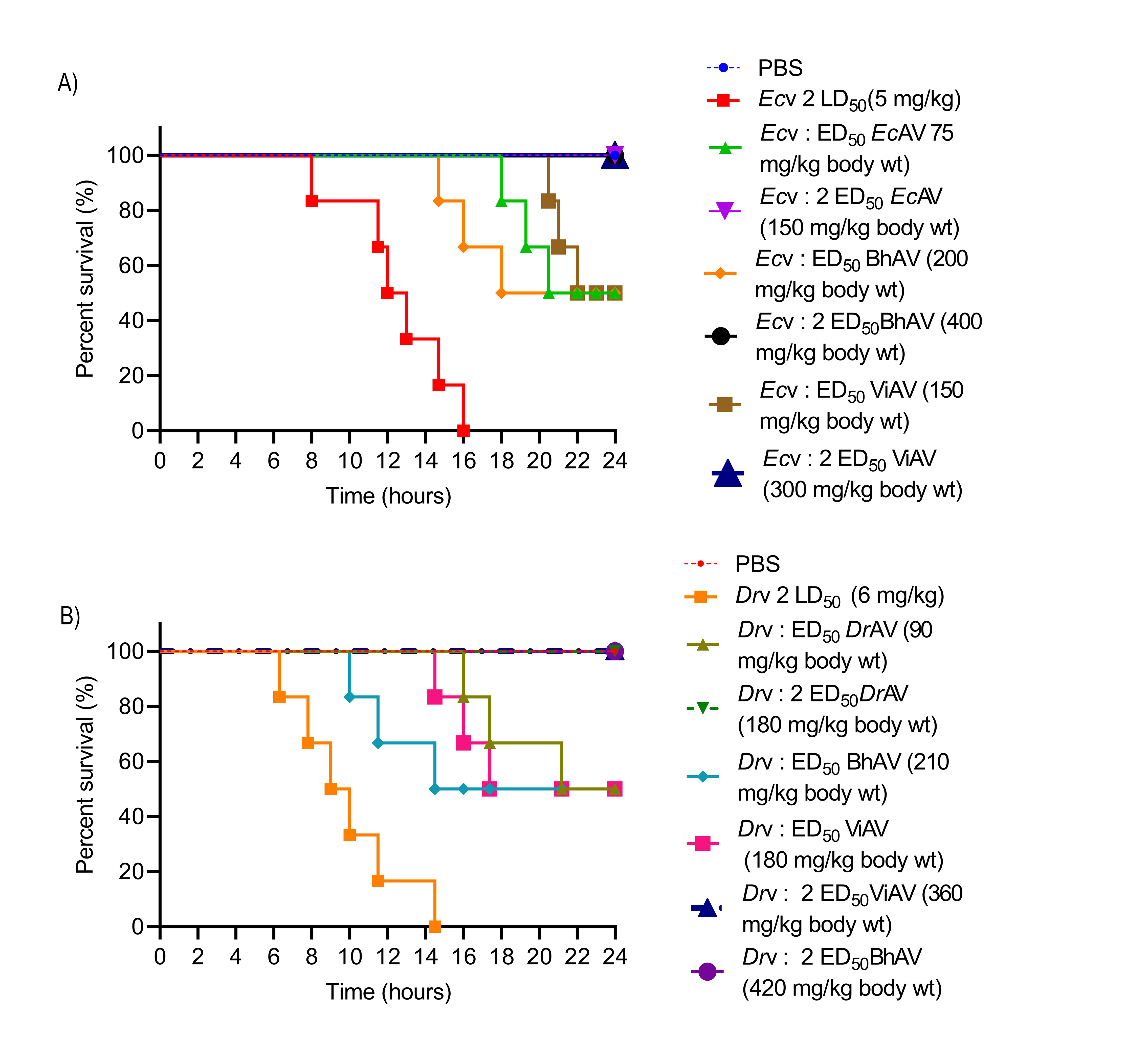

Supplement: S11 Fig — (A) Groups of mice (n = 6) were independently injected intraperitoneally (i.p.) with 2 LD50 (5 mg/kg of body weight) dose of Ecv in 50 μl PBS. Post 10 min venom injection, the mice were independently administered with 50, 75, 150, 200, 300, and 400 mg/kg of EcAV, BhAV, and ViAV respectively via mice tail vein (i.v). The effective dose (ED50) value of EcAV 75 mg/kg, BhAV 200 mg/kg, and ViAV 150 mg/kg were determined. (B) Groups of mice (n = 6) were independently injected intraperitoneally (i.p.) with 2 LD50 (5 mg/kg of body weight) dose of Drv in 50 μl PBS. Post 10 min venom injection, the mice were independently administered with 60, 90, 120, 180, 210, 360, and 420 mg/kg of DrAV, BhAV, and ViAV respectively via mice tail vein (i.v.). The effective dose value of DrAV 90 mg/kg, BhAV 240 mg/kg), and ViAV 180 mg/kg were determined. In all the cases, the groups of mice that received respective venoms alone and PBS alone were served as control experiments. Mice were kept under observation for 24 h and the time of death was recorded. The percent survival analysis of mice was done by constructing the Kaplan-Meier survival curve, the p-value was calculated using the log-rank (Mantel-Cox) test, ***p < 0.001, and **** p < 0.0001. (TIF) [file pntd.0010292.s011.tif]
